# A Discovery Strategy for Active Compounds of Chinese Medicine Based on the Prediction Model of Compound-Disease Relationship

**DOI:** 10.1155/2022/8704784

**Published:** 2022-07-08

**Authors:** Mengqi Huo, Sha Peng, Jing Li, Yanling Zhang, Yanjiang Qiao

**Affiliations:** ^1^Key Laboratory of TCM-information Engineer of State Administration of TCM, Beijing University of Chinese Medicine, Beijing, China; ^2^School of Chinese Materia Medica, Beijing University of Chinese Medicine, Beijing, China

## Abstract

An accurate characterization of diseases and compounds is the key to predicting the compound-disease relationship (CDR). However, due to the difficulty of a comprehensive description of CDR, the accuracy of traditional drug development models for large-scale CDR prediction is usually unsatisfactory. In order to solve this problem, we propose a new method that integrates the molecular descriptors of compounds and the symptom descriptors of diseases to build a CDR two-dimensional matrix to predict candidate active compounds. The Matlab software draws grayscale images of CDRs, which are used as a benchmark dataset for training convolutional neural network (CNN) models. The trained model is used to predict candidate antitumor active compounds. Among the AlexNet and GoogLeNet models, we selected the GoogLeNet model for the prediction of active compounds in Chinese medicine, and its Acc, Sen, Pre, F-measure, MCC, and AUC are 0.960, 0.956, 0.965, 0.960, 0.920, and 0.964, respectively. In the prediction results of compounds, 1624 candidate CDRs were found in 124 Chinese medicines. Among them, we obtained 31 features of candidate antitumor active compounds. This method provides new insights for the discovery of candidate active compounds in Chinese medicine.

## 1. Introduction

The accurate prediction of the compound-disease relationship (CDR) can not only be used to identify candidate active compounds but also help discover new indications for compounds. Based on the hypothesis that “compounds with similar structures tend to have similar activities”, some studies have made progress in the identification of CDRs. However, due to the lack of a comprehensive description method for compounds and diseases, the accuracy of traditional drug development models for large-scale CDRs prediction is usually unsatisfactory [1, 2].

In recent years, with the accumulation of the compound-disease relationship data, many methods have been proposed to screen candidate active compounds by identifying CDRs. These methods can be roughly divided into two major mainstreams: compound-based and disease-based. Among compound-based research methods, the quantitative structure-activity relationship (QSAR) has been widely used in the prediction of compound properties or activity [3]. The traditional QSAR method encodes molecules into fixed-length strings or vectors but ignores the detailed structure information and ADME characteristics [4]. Afterward, the holographic QSAR, 3D-QSAR, and the combination of multiple QSAR models have achieved a comprehensive description of the molecular structure of compounds. However, it is difficult to be used for the prediction of large-scale CDRs. Some molecular simulation techniques such as pharmacophore and docking can incorporate more detailed information into the calculation model, greatly improving the speed and accuracy of compound screening [5]. However, these methods usually can only obtain the effect of the compound on a target, and it is difficult to predict the overall efficacy of compounds [6]. A variety of computational models based on molecular simulation techniques have been used for the prediction and evaluation of compound-disease relationships. However, it is difficult to predict the relationship between compounds and multiple diseases for a large number of traditional Chinese medicine compounds. Based on the lack of quantitative description methods and the limitations of current computational models, this study adopted a comprehensive description method that combined molecular descriptors and disease descriptors to generate a two-dimensional matrix, which can be converted into a grayscale image that visually displays the characteristics of the data. It facilitates the identification of the relationship between large-scale compounds and multiple diseases.

For complex diseases with multiple targets, researchers have also developed a variety of disease-based methods to identify CDRs [7]. Kai Yang proposed an embedded heterogeneous network based on compound-disease relationships to predict the potential efficacy of compounds [8]. Huiqing Wang et al. combine the structural similarity of compounds with the semantic similarity of diseases, use deep learning algorithms to extract feature information, and weight features through convolution modules to effectively predict potential therapeutic drugs for diseases. [9]. However, there are still problems such as whether the data source is reliable, and the lack of relevant clinical sample data verification [10]. The above methods revolve around genes, targets, and pathways of diseases, but one of the important and neglected resources is clinical data in public literature or databases [11]. In these data, symptoms are the basis of clinical disease classification and one of the most directly related manifestations of the disease [12, 13]. Zhou et al. carried out a quantitative description of the relationship between disease and symptoms. The study standardized and weighted disease and symptom terms retrieved from large medical literature databases to build a comprehensive and high-quality disease symptom relationship map [14]. Based on the above methods, the structural information of the compound and the symptom information of the disease provide a wealth of data resources for comprehensively characterizing the compound-disease relationship.

Heat-clearing Chinese medicine plays an important role in the prevention and treatment of malignant tumors. Pharmacological studies have shown that heat-clearing Chinese medicines can achieve antitumor effects by inducing apoptosis, inhibiting the proliferation of tumor cells, regulating the immune capacity of the human body, and resisting mutations [15–17]. In addition, they also have clinical effects such as antipathogenic microorganisms, antihypertension, antiatherosclerosis, and anti-inflammation [18–20]. Heat-clearing compounds have become the research focus of new compound research and development due to their good therapeutic effects, diverse antitumor mechanisms, and few adverse reactions [21]. Among them, the pharmacological effects of some heat-clearing Chinese medicines have been deeply studied, but most of them are still in the exploratory stage. There are still many compounds with unknown efficacy yet to be developed. It needs to be screened on a large scale for better clinical application.

In recent years, deep learning has been widely used in various disciplines [22]. It is a new type of algorithm with powerful computing power, which overcomes the limitations of traditional machine learning, such as weak data adaptability and less applicable data types [23]. Among them, Convolutional Neural Network (CNN) is one of the deep learning algorithms that can be used in image recognition. It has the advantages of local connections, weight sharing, and pooling, which can reduce the parameters in the network. The structural features of network local connection and convolution kernel weight sharing reduce the complexity of the model, especially when dealing with two-dimensional images, it has good robustness for image displacement, scaling, and nonlinear deformation [24]. These features make the CNN model have high computational efficiency and classification accuracy [25].

AlexNet is one of the common architectures of the CNN model. Research has shown that the more layers of the CNN model, the higher the accuracy. However, due to the problem of the disappearance of the gradient, the gradient becomes smaller in the back propagation process, making the weight unable to be updated. Therefore, CNN performance will not completely increase as the number of layers increases. GoogLeNet solves this problem very well. The modular structure of inception makes it increase the depth and width of the network without increasing the parameters [26]. Therefore, it can achieve better prediction performance with fewer parameters. It is suitable for complex and multidimensional input data.

**Figure 1:**
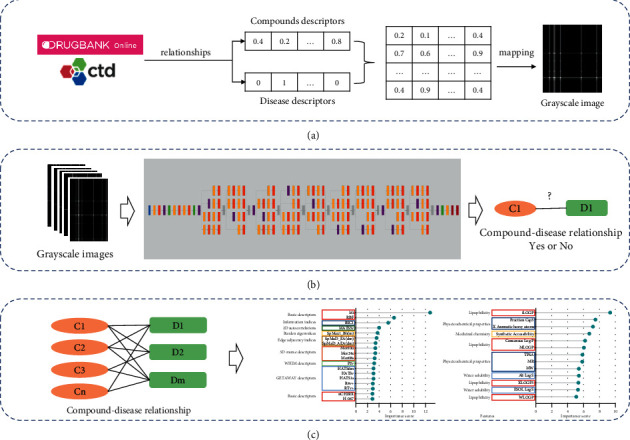
The experimental flow chart. (a) Draw a grayscale map based on the existing compound-disease relationship (CDR). (b) Convolutional Neural Networks (CNNs) are used to build models that identify candidate CDRs. (c) Models are used to predict candidate active compounds and extract structural features of antitumor compounds.

Therefore, based on the structural information of the compound and the information on the symptoms of the disease, this study established a description method of disease and compound characterization. We use a combination of molecular descriptors and symptom descriptors to establish a CDR two-dimensional matrix. The CDRs contained in public databases are mapped to grayscale images. These images are used as a benchmark data set to train the CNN model. Among the AlexNet and GoogLeNet models, we selected the GoogLeNet model for the prediction of active compounds in traditional Chinese medicine. Among them, we obtained 31 features of candidate antitumor active compounds. This method can provide a new perspective for the large-scale development of active compounds in traditional Chinese medicine. The experimental flowchart is shown in Figure 1.

## 2. Results

From the Comparative Toxicogenomics Database (CTD, version 1.0 http://ctdbase.org/, accessed July 15, 2021) and Drugbank database (version 5.1.9, https://go.compoundbank.com/, accessed July 14, 2021), we collected a total of 13981 confirmed CDRs as positive samples, including 40 diseases and 4770 compounds [27, 28]. However, due to the lack of research value, there is currently no database dedicated to the inclusion of unrelated compounds and diseases. We had to randomly select 13981 unknown compound-disease relationships (NCDRs) as negative samples from 176819 unrelated compounds and diseases; CDRs are not included in these NCDR data and used them together with positive samples as the benchmark data set.

### 2.1. Proportion of Positive and Negative Samples

**Figure 2:**
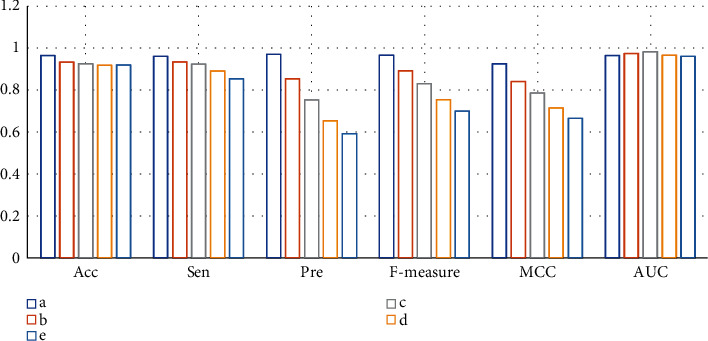
The calculation results of the test set with positive and negative sample ratios set to 1 : 1(a), 1 : 2(b), 1 : 3(c), 1 : 4(d), and 1 : 5(e).

In CDRs composed of compounds and diseases, the number of negative samples is far more than that of positive samples. To overcome the common unbalanced problem of the data set in classification models, we try to examine the proportion of positive and negative samples in the benchmark dataset. A series of datasets were constructed, with positive and negative sample ratios of 1 : 1, 1 : 2, 1 : 3, 1 : 4, and 1 : 5, respectively. 20,000 samples are randomly selected from the benchmark dataset, 75% of which are used to generate the training set, and the remaining 25% are used to generate the test set. The whole process is repeated three times, and the average statistical results of the test set are shown in Figure 2.

In the test set, the ratio of positive and negative samples is from 1 : 1 to 1 : 5. The results showed that the Acc and AUC values of the model did not change much. However, the values of Sen, Pre, F-measure, and MCC all decreased significantly as the ratio increased. These results show that as the ratio of negative samples increases, the number of negative samples in the training set also increases. The model is more fully trained on negative samples, so it is easier to identify negative samples, but it is also harder to identify positive samples. This suggests that it is reasonable to set the ratio of positive and negative samples to 1 : 1, which can ensure that the model has high sensitivity.

### 2.2. Prediction Results of GoogLeNet Model and AlexNet Model

**Figure 3:**
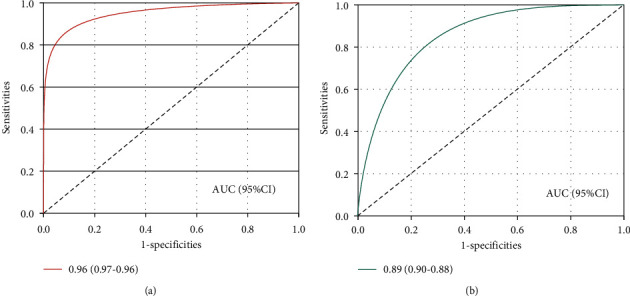
ROC curves of GoogLeNet and AlexNet models. (a) ROC curves of the GoogLeNet model. (b) ROC curves of the AlexNet model.

In order to select a more suitable CNN model, we used the same benchmark dataset to train and test the GoogLeNet model and the AlexNet model. 20,000 samples are randomly selected from the benchmark dataset, 75% of which are used to generate the training set, and the remaining 25% are used to generate the test set, ensuring that the number of positive and negative samples remains the same. Based on the same training set, the GoogLeNet model and the AlexNet model are trained separately. Then, we use the same test set to measure the performance of the two models. The results are shown in Table 1 and Figure 3.

Since the two models are both binary classification models, we compared their ability to recognize positive and negative samples, respectively (Table 2). In terms of the ability to identify positive samples, the Sen, Pre, and F-measure of the GoogLeNet model are 0.956, 0.965, and 0.960, which are 2.465%, 6.866%, and 4.575% higher than the AlexNet model, respectively. In terms of the ability to identify negative samples, the Sen, Pre, and F-measure of the GoogLeNet model are 0.964, 0.956, and 0.960, which are 6.402%, 2.137%, and 4.234% higher than the AlexNet model, respectively. Overall, among the two models, the GoogLeNet model is more suitable for our experimental data.

### 2.3. Identiﬁcation Power of the GoogLeNet Model on the External Test Set

#### 2.3.1. The Ability of the Model to Identify CDRs for Existing Compounds and New Indications

We further evaluated the recognition ability of the GoogLeNet model for a variety of external test sets. By generating new training sets and test sets, the predictive ability of the GoogLeNet model for existing compounds and new indications is evaluated. (1) Randomly select a positive sample (The CDR contains compound *C*_m_ and disease *D*_i_ ) from the benchmark data set to the training set. (2) All positive samples including disease *D*_i_ were put into the training set. (3) Repeat (1) and (2) until the number of positive samples in the training set reaches 7500, and 2500 of the remaining positive samples are selected as the test set. (4) Randomly select a negative sample (The NCDR contains compound *C*_n_ and disease *D*_i_) from the benchmark data set to the training set. (5) All negative samples including disease *D*_i_ are put into the training set. (6) Repeat (1) and (2) until the number of negative samples in the training set reaches 7500, and 2500 of the remaining negative samples are selected as the test set.

**Figure 4:**
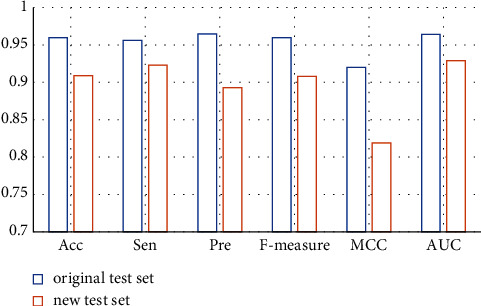
The prediction results of the GoogLeNet model on the new test set and the original test set.

Based on this strategy, it can be ensured that disease information in the test set does not exist in the training set. The prediction result is shown in Figure 4.

In the new test set, Acc, Sen, Pre, F-measure, MCC and AUC are 0.909, 0.923, 0.893, 0.908, 0.819, and 0.929, respectively, which are about 5.312%, 3.452%, 7.461%, 5.417%, 10.978%, and 3.631% lower than the test set of the original benchmark data set. These results show that even though the disease in the test set does not exist in the training set, our method can still obtain high prediction accuracy. The model has the ability to identify new indications for compounds.

#### 2.3.2. Recognition Ability of Candidate Compound Molecules

**Figure 5:**
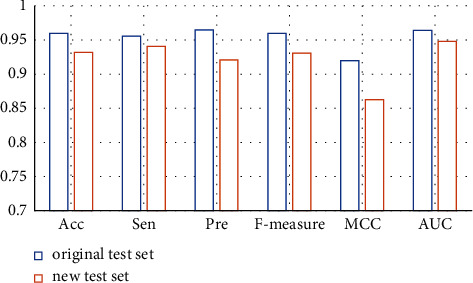
The prediction results of the GoogLeNet model on the new test set and the original test set.

Similarly, by constructing new training sets and test sets, we further evaluated the performance of the model in identifying relationships between existing diseases and new compounds. Following the above strategy, it was ensured that compounds in the test set were not present in the training set. The predicted results are shown in Figure 5.

In the new test set, Acc, Sen, Pre, F-measure, MCC, and AUC are 0.932, 0.941, 0.921, 0.931, 0.863, and 0.948, respectively. It is only about 2.917%, 1.569%, 4.560%, 3.021%, 6.196%, and 1.660% lower than the test set of the original benchmark data set. These results show that the model can identify candidate compounds with high prediction accuracy.

### 2.4. Large-Scale Prediction Results of Heat-Clearing Chinese Medicine Compounds

There are a total of 124 Chinese medicines classified as heat-clearing Chinese medicines in the SymMap database (version 2.0, http://www.symmap.org/, accessed May 26, 2021), and 2302 compounds of them are downloaded [29]. Chinese medicine compounds are annotated in the SymMap database. QC compounds refer to critical compounds under the quality inspection of herbs. Blood compounds refer to compounds absorbed into the blood and can be detected in the blood. We only select QC compounds, blood compounds, and compounds with OB>30% [30–32]. After filtering, 83 Chinese medicines and 437 compounds are retained. Finally, 437 compounds and 40 diseases generated 17480 unknown CDRs. The model predicted 1624 candidate CDRs. We sort all CDRs in descending order according to the probability value. Among them, 1416 CDR probability values are greater than 0.9, and these compounds may become candidate active compounds (Table S1).

### 2.5. Structural Features and ADME Features of Antitumor Active Compounds

**Figure 6:**
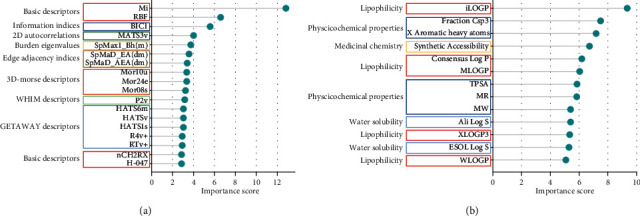
The feature importance score of antitumor compounds based on SVM-RFE. (a) The structure feature importance score of antitumor compounds, red boxes represent basic descriptors, the blue box represents information indices, the dark green box represents 2D autocorrelations, the yellow box represents burden eigenvalues, the brown box represents edge adjacency indices, the red-brown box represents3D-MoRSE descriptors, the green box represents WHIM descriptors, and the sky blue box represents GETAWAY descriptors. (b) The ADME feature importance score of antitumor compounds, red boxes represent lipophilicity, blue boxes represent physicochemical properties, the yellow box represents medicinal chemistry, and sky blue boxes represent water solubility.

In order to discover the structure and ADME feature of candidate antitumor compounds, we use the support vector machine recursive feature elimination (SVM-RFE) algorithm to calculate the feature importance scores for their molecular descriptors and ADME descriptors. In the end, we obtained 31 features of antitumor compounds including 13 ADME features and 18 structure features. The result is shown in Figure 6 and Table S2.

There are 30 categories of molecular structure features in the Dragon software, including ring descriptor, topological index descriptor, geometric descriptor, and pharmacokinetic index descriptor. As can be seen in Figure 6(a), the features with the highest scores are basic descriptors (Mi, RBF, nCH2RX, H-047), followed by information indices (BIC1), 2D autocorrelations (MATS3v), burden eigenvalues (SpMax1_Bh(m)), edge adjacency indices (SpMAD_EA(dm), SpMAD_AEA(dm)), 3D-MoRSE descriptors (Mor10u, Mor24e, Mor08s), WHIM descriptors(P2v), and GETAWAY descriptors(HATS6m, HATSv, HATS1s, R4v+, RTv+).

There are 44 ADME descriptors in the SwissADME database, including 6 compound-likeness, 6 lipophilicity, 4 medicinal chemistry, 9 pharmacokinetics, 10 physicochemical properties, and 9 water solubility. As can be seen in Figure 6(b), the feature with the highest scores is lipophilicity (iLOGP, Consensus Log P, MLOGP, XLOGP3, WLOGP), followed by physicochemical properties (Fraction Csp3, *X* Aromatic heavy atoms, TPSA, MR, MW), medicinal chemistry (synthetic accessibility), and water solubility (Ali Log S, ESOL Log S).

## 3. Discussion

In this study, we used symptom descriptors of diseases and molecular descriptors of compounds to draw novel two-dimensional matrix grayscales. The CNN algorithm is used to build a prediction model to identify candidate CDRs. The rationality of this method stems from the fact that “structure determines function” and “symptoms are clinical manifestations of disease” [11]. Therefore, we use molecular descriptors to describe compound structure information and symptom descriptors to represent the pathological mechanism of the disease. This method provides new insights for the discovery of candidate active compounds.

In the process of model construction, we first examine the impact of the sample unbalance problem on model prediction performance. Because of CDRs that we can actually obtain, negative samples are far more than positive samples. When the positive and negative sample ratios were set to 1 : 1, 1 : 2, 1 : 3, 1 : 4, and 1 : 5, the values of Pre, Sen, F-measure, and MCC all dropped significantly. However, Acc has not changed much. This is because Acc considers the combined results of positive and negative samples. When there are too many negative samples in the training set, the value of Acc is mainly determined by the negative samples. Since the training set provides more negative sample information, it will also increase the accuracy of the model in identifying negative samples. Therefore, Acc has not changed much with the increase in the ratio. In addition, we have also noticed that in the current model, when the number of positive and negative samples was changed, the AUC did not decrease significantly.

After that, we compared the prediction results of the AlexNet model and the GoogLeNet model for CDRs. In the same test set, the GoogLeNet model is more suitable for our experimental data. Therefore, we identified the predictive ability of the GoogLeNet model on the external test set. Discovering new indications for compounds can greatly reduce the time and money invested in drug development, so many researchers have turned their attention to “new use of old compounds.” We added new diseases to the test set, and the model can still obtain a high prediction accuracy. This indicates that the model has the ability to identify new indications for existing compounds. Researchers usually pay more attention to which compounds may become candidate lead compounds, which is a key step in the design and optimization of new drugs. We added new compounds to the test set. The results show that the model has the ability to identify candidate active compounds.

Some previous reports can be used to verify our prediction results. In CDRs predicted by the model, the antitumor effects of some compounds have been reported. For example, the model predicts that eugenol, eucalyptol, eupatorin, shikonin, jatrorrhizine, and isovitexin have candidate antitumor activities. Studies have shown that eugenol can produce anticancer effects by blocking the cell cycle, inducing cell death, and inhibiting tumor cell metastasis. [33–35]. In addition, eugenol can reduce the toxicity of chemotherapy and improve its efficacy and can be used as an adjuvant drug for chemotherapy. [36]. Eugenol inhibits tumor migration and invasion by regulating the PI3K/Akt/mTOR pathway [37]. A pharmacological study showed that eupatorin (20 mg/kg) was able to reduce tumor lung metastases compared to untreated mice. [38]. The study found that shikonin can significantly inhibit the proliferation of hepatoma cells (HepG2, BEL7402, and Huh7). However, it has no obvious inhibitory effect on normal human hepatocytes cells (L02) and human embryonic kidney cells (HEK293T), suggesting that shikonin can selectively kill tumor cells [39]. The inhibition of tumor cell proliferation by shikonin is closely related to its inhibition of the cell cycle. Studies have found that shikonin can arrest gastric cancer cells (AGS) in the G2/M phase, hepatoma cells (HepG2) in the S phase, and colon cancer cells (HCT116 and SW480) in the G1 phase. Shikonin arrests the cycle of tumor cells by regulating the expression of cycle-related proteins, such as cyclin A, cyclin B, CDK1, and CDK2 [40]. Jatrorrhizine can reduce *β*-catenin and increase GSK-3*β* expression thereby inhibiting the epithelial-mesenchymal transition of cells. A pharmacological study showed that jatrorrhizine can induce tumor cell apoptosis and inhibit tumor growth and metastasis in the HCT-116 nude mouse xenograft model [41]. Isovitexin induces tumor cell apoptosis and inhibits colon cancer cell growth through PI3K/Akt/mTOR signaling pathway [42]. There are also some candidate antitumor compounds predicted by models that have not been found to have relevant efficacy in previous studies. For example, yadanzioside B, beta-hydroxypropiovanillone, dihydrokaempferide, precatorine, and so on. They may become candidates for future compound design directions.

Traditional compound efficacy prediction models are usually based on a single type of compounds or a single target, which is difficult to use for large-scale compound-disease relationship prediction. When new compounds or new indications appear, traditional compound efficacy prediction models will be unsatisfactory [26]. In this study, based on the GoogLeNet algorithm, a CDRs prediction model was constructed for more than 4000 compounds in the CTD database and the Drugbank database. Taking heat-clearing Chinese medicine as an example, we apply the model to the large-scale prediction of compounds' efficacy. The model can obtain large quantities of compound-disease relationship data through one calculation. It provides a data foundation for the construction of a big data platform for traditional Chinese medicine and also provides technical support for the mechanism study of multicompounds-multitargets of traditional Chinese medicine.

This research has certain limitations. For example, we only selected 40 common clinical diseases as the research objects. In fact, the classification of commonly clinical diseases and the use of compounds are more abundant and diversified. We have only made a methodological attempt in this research. In future research, we will expand the benchmark data set and refine the disease classification. In addition, when predicting diseases related to Chinese medicine compounds, we only used QC compounds, blood compounds, and compounds with OB>30%. In fact, it is still a huge challenge to study how Chinese medicine compounds enter the body to be absorbed and metabolized [43]. With the establishment of more traditional Chinese medicine compounds databases and the identification of blood compounds, the number of compounds predicted by the model will also increase. There will also be more potential active compounds of traditional Chinese medicine to be screened for the design of new drugs. Finally, the candidate active compounds predicted by the model still need to be confirmed in future studies.

## 4. Materials and Methods

### 4.1. Collection of Compound-Disease Relationship Data

The human CDR was downloaded from the CTD database and Drugbank database. We only used compounds with “direct evidence (marker/mechanism/treatment)” annotation, which indicates the accurate relationship between disease and compounds [11].

For compounds, we calculated their 5270 structure descriptors through the Dragon 7 software (Talete Srl, Milano, Italy). It can obtain chemical structure information including atom type, functional group, number of fragments, topology, and geometric descriptors [44]. Therefore, each compound can be described by a 5270-dimensional feature vector.

For diseases, we retrieved the symptom data of 40 diseases from the human symptom-disease network. The study is based on the cooccurrence of diseases and symptoms in the PubMed database to obtain the relationship between them. All diseases can be represented by 322 symptom descriptors [14]. Therefore, each disease can be described by a 322-dimensional feature vector. The molecular descriptor and symptom descriptor are normalized separately.

### 4.2. Plotting of Grayscale Map of Compound-Disease Relationship Matrix

Each CDR is mapped into a two-dimensional matrix. Then, they are converted into grayscale images in order to train the CNN model. First, standardize the color block range of the grayscale image to 0–255. Then, for each CDR, we use the disease feature as the row and the compound feature as the column and calculate the average of the sums of each row and corresponding column. Finally, a two-dimensional relationship matrix of diseases and compounds of size 5270 × 322 can be obtained. That is, the disease is represented by the feature vector *V*_D_ = [*a*1, *a*2, ..., *a*322]. For the compound, its characteristic vector is *V*_C_ = [*b*1, *b*2, ..., *b*5270]. For a CDR, it is represented by a matrix *M*, where the elements in the j-th row and the i-th column are (aj + bi)/2. The matrix M is converted into a grayscale image by MATLAB 2019a software (Mathworks, Natick, MA) to represent a CDR.

(1) $$\begin{array}{c} M_{ij}=\frac{\left(a_{j}+b_{i}\right)}{2}. \end{array}$$

### 4.3. Construction of GoogLeNet Model and AlexNet Model

**Figure 7:**
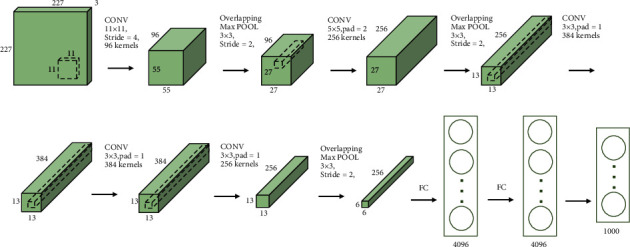
The network structure of the AlexNet model.

**Figure 8:**
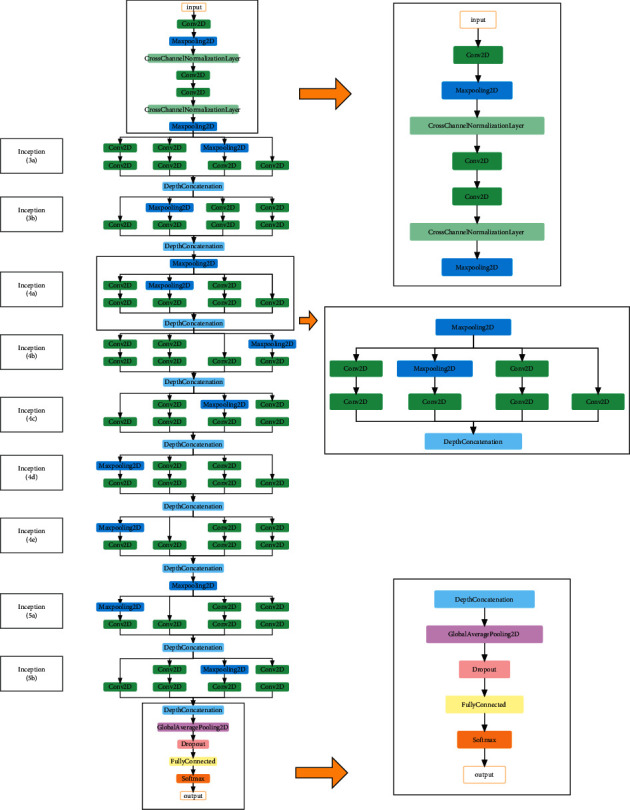
The network structure of the GoogLeNet model.

The purpose of the model is to judge the yes or no of each CDR, which is a binary classification problem. The AlexNet model is divided into eight layers. After five layers of convolution operations, it is connected to three layers of fully connected layers. Compared with the traditional CNN model, the AlexNet model has a deep network structure and many parameters and has stronger feature expression capabilities. It uses RELU as the activation function, which improves the computational efficiency of the network. The network structure is shown in Figure 7 (Table S3) [45]. The GoogLeNet model is a deep CNN model. The structure of the model is shown in Figure 8 (Table S4). It reduces the number of parameters and improves computational efficiency by using the inception module [46]. The momentum of the model stochastic gradient descent optimizer is set to 0.9 and the learning rate is set to 0.0001. The maximum number of training epochs was set to 20 epochs with 290 iterations per epoch, using a small batch of 20 observations in each iteration. All other parameters use default values. The program is executed based on MATLAB 2019a software.


(2) $$\begin{array}{c} Acc=\frac{TP+TN}{TP+TN+FP+FN}, \end{array}$$

(3) $$\begin{array}{c} \text{Pre}=\frac{TP}{TP+FP}, \end{array}$$

(4) $$\begin{array}{c} \text{Sen}=\frac{TP}{TP+FN}, \end{array}$$

(5) $$\begin{array}{c} F-\text{measure}=\frac{2TP}{2TP+FP+FN}, \end{array}$$

(6) $$\begin{array}{c} \text{MCC}=\frac{TP\times TN-FP\times FN}{\sqrt{\left(TP+FP\right)\left(TP+FN\right)\left(TN+FN\right)}}. \end{array}$$

We tested the GoogLeNet model and the AlexNet model using the same benchmark dataset. 20000 samples were randomly drawn from the benchmark dataset, with the same number of positive and negative samples. We select 75% of them to generate the training set and the remaining 25% to generate the test set. Accuracy (Acc), sensitivity (Sen), precision (Pre), Matthews correlation coefficient (MCC), F-measure, and AUC are commonly used evaluation indicators in binary classification models. The calculation results of the above evaluation indicators were used to select the CNN model suitable for this study.

Here, true positives (TPs) represent the number of samples that were predicted to be positive and were actually positive. False positives (FP) represent the number of samples that were predicted to be positive and were actually negative. True negatives (TNs) represent the number of samples predicted to be negative and actually negative. False negatives (FNs) represent the number of samples predicted to be negative and actually positive.

### 4.4. The Large-Scale Prediction of Heat-Clearing Chinese Medicine Compounds

The trained GoogLeNet model was applied to the large-scale prediction of heat-clearing Chinese medicines compounds. The GoogLeNet model attempts to discover candidate indications for their compounds. In order to generate unknown CDRs, we (1) downloaded the 3D structure of the heat-clearing Chinese medicine compounds from the SymMap datasets, (2) only select the compounds marked as QC compounds, blood compounds, and OB>30% in the database (Table S5), and (3) randomly combine compounds with 40 diseases. Follow steps 4.1 and 4.2 to generate grayscale images of CDRs. They are input into the trained model to predict the relationship between compounds and diseases.

### 4.5. Structural Features and ADME Features' Extraction of Antitumor Compounds Based on SVM-RFE Algorithm

The predicted CDRs were used to extract the structural features and ADME features of the candidate antitumor compounds. We enter the SMILES of antitumor compounds into the SwissADME database and can obtain 44 ADME descriptors (version 1.0, http://www.swissadme.ch/, accessed August 16, 2021) [47]. We employ the SVM-RFE algorithm to calculate feature importance scores for antitumor compounds. The SVM-RFE algorithm is a selection algorithm for sorting all features based on the SVM model. It uses w^2^ to represent the importance of features as the basis for feature ranking. The algorithm starts with all features and then removes one feature with the smallest w^2^ at a time until the feature set is empty. The program is implemented based on *R* 4.0.2 software (https://www.r-project.org/). Each feature importance score *F*_i_ can be expressed as

(7) $$\begin{array}{c} F_{i}=w_{i}^{2}. \end{array}$$

Here, w is the feature weight vector of the SVM model; w_i_ is the weight of the i-th dimension feature [48].

## Figures and Tables

**Table 1:** Calculation results of the test set based on the GoogLeNet model and the AlexNet model.

CNN	Acc	Sen	Pre	F-measure	MCC	AUC
GoogLeNet	0.960	0.956	0.965	0.960	0.920	0.964
AlexNet	0.919	0.933	0.903	0.918	0.839	0.889

In the same test set, GoogLeNet shows an AUC of 0.964; the AlexNet model shows an AUC of 0.889. Furthermore, the GoogLeNet model has higher values in terms of Acc, Sen, Pre, F-measure, and MCC. It outperforms the AlexNet model by 4.461%, 2.465%, 6.866%, 4.575%, 9.654%, and 8.436%, respectively.

**Table 2:** Calculation results of positive and negative samples based on the GoogLeNet and AlexNet models.

	Sen	Pre	F-measure	Class
GoogLeNet	0.956	0.965	0.960	1
AlexNet	0.933	0.903	0.918	1
GoogLeNet	0.964	0.956	0.960	0
AlexNet	0.906	0.936	0.921	0

## Data Availability

The data used to support the findings of this study are available from the corresponding author upon request.

## Supplementary Materials

Supplementary Materials Supplementary Materials. Table S1: prediction results of compounds of Chinese medicine. Table S2: importance score of antitumor compound features. Table S3: the network structure of the AlexNet model. Table S4: the network structure of the GoogLeNet model. Table S5: compounds of heat-clearing Chinese medicines in the SymMap database.
