# Supplementary material for: A Discovery Strategy for Active Compounds of Chinese Medicine Based on the Prediction Model of Compound-Disease Relationship
Source: J Oncol. 2022 Jul 8;2022:8704784. doi: 10.1155/2022/8704784 (PMC9286898; doi:10.1155/2022/8704784)
Supplement: Supplementary Materials — Table S1: prediction results of compounds of Chinese medicine. Table S2: importance score of antitumor compound features. Table S3: the network structure of the AlexNet model. Table S4: the network structure of the GoogLeNet model. Table S5: compounds of heat-clearing Chinese medicines in the SymMap database. [file 8704784.f1.zip › Table S1.docx]

Table S1: Prediction results of compounds of Chinese medicine.

| compounds | diseases | value |
| --- | --- | --- |
| 753 | Fever | 1.000 |
| 846 | Parkinson Disease | 1.000 |
| 938 | Rheumatic Diseases | 1.000 |
| 1174 | Virus Diseases | 1.000 |
| 1183 | Rheumatic Diseases | 1.000 |
| 5610 | Hyperlipidemias | 1.000 |
| 6736 | Hyperlipidemias | 1.000 |
| 11468 | Fever | 1.000 |
| 12127 | Atherosclerosis | 1.000 |
| 13603 | Gonadal Dysgenesis | 1.000 |
| 16928 | Hyperlipidemias | 1.000 |
| 23752 | Inflammation | 1.000 |
| 62065 | Parkinson Disease | 1.000 |
| 68081 | Hypersensitivity | 1.000 |
| 72953 | Virus Diseases | 1.000 |
| 79022 | Atherosclerosis | 1.000 |
| 81722 | Immunologic Deficiency Syndromes | 1.000 |
| 98914 | Mycoses | 1.000 |
| 99474 | Schizophrenia | 1.000 |
| 100332 | Neoplasm | 1.000 |
| 107217 | Inflammation | 1.000 |
| 119034 | Hypersensitivity | 1.000 |
| 119258 | Bacterial Infections | 1.000 |
| 122325 | Hypotension | 1.000 |
| 145858 | Schizophrenia | 1.000 |
| 185914 | Atherosclerosis | 1.000 |
| 381152 | Mycoses | 1.000 |
| 443716 | Edema | 1.000 |
| 443716 | Inflammation | 1.000 |
| 445858 | Virus Diseases | 1.000 |
| 521300 | Cholestasis | 1.000 |
| 637511 | Parkinson Disease | 1.000 |
| 1549018 | Thrombosis | 1.000 |
| 1549106 | Inflammation | 1.000 |
| 3080635 | Parkinson Disease | 1.000 |
| 3084331 | Intestinal Diseases, Parasitic | 1.000 |
| 5280448 | Hyperlipidemias | 1.000 |
| 5280544 | Inflammation | 1.000 |
| 5280794 | Edema | 1.000 |
| 5280794 | Fever | 1.000 |
| 5280863 | Edema | 1.000 |
| 5280863 | Hypertension | 1.000 |
| 5280933 | Edema | 1.000 |
| 5280933 | Hypertension | 1.000 |
| 5281325 | Angina Pectoris | 1.000 |
| 5281703 | Inflammation | 1.000 |
| 5318889 | Immunologic Deficiency Syndromes | 1.000 |
| 5318889 | Virus Diseases | 1.000 |
| 5364509 | Immunologic Deficiency Syndromes | 1.000 |
| 5488781 | Gonadal Dysgenesis | 1.000 |
| 5488781 | Infertility, Female | 1.000 |
| 5491637 | Parkinson Disease | 1.000 |
| 6432468 | Stomach Ulcer | 1.000 |
| 9601691 | Hypertension | 1.000 |
| 9601691 | Inflammation | 1.000 |
| 9601691 | Liver Cirrhosis | 1.000 |
| 10899740 | Hypotension | 1.000 |
| 11250133 | Angina Pectoris | 1.000 |
| 14656909 | Hypotension | 1.000 |
| 14849116 | Renal Insufficiency, Chronic | 1.000 |
| 44257585 | Hypotension | 1.000 |
| 71308174 | Liver Cirrhosis | 1.000 |
| 73817508 | Fever | 1.000 |
| 119058017 | Epilepsy | 1.000 |
| 311 | Neoplasm | 0.999 |
| 335 | Virus Diseases | 0.999 |
| 3218 | Edema | 0.999 |
| 3314 | Neoplasm | 0.999 |
| 4680 | Epilepsy | 0.999 |
| 6199 | Hemorrhage | 0.999 |
| 6274 | Inflammation | 0.999 |
| 7302 | Neoplasm | 0.999 |
| 7809 | Immunologic Deficiency Syndromes | 0.999 |
| 9750 | Hypertension | 0.999 |
| 10207 | Cholestasis | 0.999 |
| 10228 | Epilepsy | 0.999 |
| 11468 | Cholestasis | 0.999 |
| 12516 | Hypotension | 0.999 |
| 13603 | Epilepsy | 0.999 |
| 19009 | Inflammation | 0.999 |
| 19009 | Neoplasm | 0.999 |
| 62065 | Epilepsy | 0.999 |
| 68081 | Hyperlipidemias | 0.999 |
| 68085 | Inflammation | 0.999 |
| 72322 | Inflammation | 0.999 |
| 72323 | Diabetes Mellitus | 0.999 |
| 73299 | Inflammation | 0.999 |
| 75142 | Virus Diseases | 0.999 |
| 82755 | Neoplasm | 0.999 |
| 99474 | Hypertension | 0.999 |
| 119034 | Osteoporosis | 0.999 |
| 145858 | Neoplasm | 0.999 |
| 155331 | Bacterial Infections | 0.999 |
| 156051 | Angina Pectoris | 0.999 |
| 160876 | Hyperlipidemias | 0.999 |
| 161294 | Asthma | 0.999 |
| 163040 | Liver Cirrhosis | 0.999 |
| 169494 | Cholestasis | 0.999 |
| 188316 | Inflammation | 0.999 |
| 222064 | Hyperlipidemias | 0.999 |
| 222284 | Cough | 0.999 |
| 222284 | Neoplasm | 0.999 |
| 285698 | Inflammation | 0.999 |
| 441737 | Neoplasm | 0.999 |
| 442408 | Infertility, Female | 0.999 |
| 442408 | Inflammation | 0.999 |
| 442688 | Inflammation | 0.999 |
| 521300 | Fever | 0.999 |
| 586387 | Inflammation | 0.999 |
| 637511 | Bacterial Infections | 0.999 |
| 637511 | Mycoses | 0.999 |
| 638072 | Atherosclerosis | 0.999 |
| 638072 | Hypertension | 0.999 |
| 1548910 | Parkinson Disease | 0.999 |
| 1549025 | Intestinal Diseases, Parasitic | 0.999 |
| 5280343 | Virus Diseases | 0.999 |
| 5280378 | Schizophrenia | 0.999 |
| 5280442 | Atherosclerosis | 0.999 |
| 5280442 | Hypertension | 0.999 |
| 5280445 | Parkinson Disease | 0.999 |
| 5280489 | Epilepsy | 0.999 |
| 5280537 | Angina Pectoris | 0.999 |
| 5280537 | Fever | 0.999 |
| 5280794 | Inflammation | 0.999 |
| 5280794 | Renal Insufficiency, Chronic | 0.999 |
| 5280862 | Bacterial Infections | 0.999 |
| 5280933 | Neoplasm | 0.999 |
| 5281232 | Atherosclerosis | 0.999 |
| 5281331 | Bacterial Infections | 0.999 |
| 5281331 | Epilepsy | 0.999 |
| 5281654 | Inflammation | 0.999 |
| 5281654 | Neoplasm | 0.999 |
| 5281707 | Cholestasis | 0.999 |
| 5283316 | Immunologic Deficiency Syndromes | 0.999 |
| 5317205 | Thrombosis | 0.999 |
| 5317360 | Immunologic Deficiency Syndromes | 0.999 |
| 5318042 | Inflammation | 0.999 |
| 5318565 | Schizophrenia | 0.999 |
| 5320351 | Mycoses | 0.999 |
| 5320438 | Inflammation | 0.999 |
| 5321018 | Inflammation | 0.999 |
| 5321047 | Liver Cirrhosis | 0.999 |
| 5321977 | Inflammation | 0.999 |
| 5322059 | Neoplasm | 0.999 |
| 5354264 | Virus Diseases | 0.999 |
| 5366074 | Inflammation | 0.999 |
| 5372954 | Bacterial Infections | 0.999 |
| 5372954 | Epilepsy | 0.999 |
| 5471349 | Neoplasm | 0.999 |
| 6433206 | Hyperlipidemias | 0.999 |
| 6540490 | Bacterial Infections | 0.999 |
| 9889962 | Angina Pectoris | 0.999 |
| 10026486 | Inflammation | 0.999 |
| 10899740 | Edema | 0.999 |
| 11600642 | Atherosclerosis | 0.999 |
| 11982272 | Liver Cirrhosis | 0.999 |
| 12302744 | Immunologic Deficiency Syndromes | 0.999 |
| 21117640 | Hypertension | 0.999 |
| 46173956 | Immunologic Deficiency Syndromes | 0.999 |
| 54711004 | Bacterial Infections | 0.999 |
| 71306914 | Schizophrenia | 0.999 |
| 101937309 | Nephritis | 0.999 |
| 131675179 | Hypertension | 0.999 |
| 146158060 | Epilepsy | 0.999 |
| 247 | Neoplasm | 0.998 |
| 338 | Fever | 0.998 |
| 460 | Bacterial Infections | 0.998 |
| 938 | Gout | 0.998 |
| 1174 | Diabetes Mellitus | 0.998 |
| 6027 | Bacterial Infections | 0.998 |
| 6199 | Epilepsy | 0.998 |
| 6274 | Diabetes Mellitus | 0.998 |
| 6322 | Angina Pectoris | 0.998 |
| 6448 | Inflammation | 0.998 |
| 6549 | Edema | 0.998 |
| 7041 | Immunologic Deficiency Syndromes | 0.998 |
| 7150 | Intestinal Diseases, Parasitic | 0.998 |
| 9015 | Hypertension | 0.998 |
| 9064 | Liver Cirrhosis | 0.998 |
| 10207 | Fever | 0.998 |
| 10228 | Bacterial Infections | 0.998 |
| 10228 | Thrombosis | 0.998 |
| 10430 | Virus Diseases | 0.998 |
| 10795 | Fever | 0.998 |
| 10795 | Inflammation | 0.998 |
| 16066 | Immunologic Deficiency Syndromes | 0.998 |
| 16724 | Fever | 0.998 |
| 16928 | Atherosclerosis | 0.998 |
| 62065 | Schizophrenia | 0.998 |
| 68081 | Hypertension | 0.998 |
| 71082 | Immunologic Deficiency Syndromes | 0.998 |
| 72344 | Inflammation | 0.998 |
| 72344 | Liver Cirrhosis | 0.998 |
| 72961 | Neoplasm | 0.998 |
| 76851 | Thrombosis | 0.998 |
| 81722 | Inflammation | 0.998 |
| 94162 | Inflammation | 0.998 |
| 99474 | Edema | 0.998 |
| 99474 | Renal Insufficiency, Chronic | 0.998 |
| 100332 | Atherosclerosis | 0.998 |
| 100332 | Liver Cirrhosis | 0.998 |
| 119034 | Edema | 0.998 |
| 124069 | Inflammation | 0.998 |
| 145858 | Atherosclerosis | 0.998 |
| 145858 | Parkinson Disease | 0.998 |
| 145937 | Liver Cirrhosis | 0.998 |
| 160876 | Angina Pectoris | 0.998 |
| 160876 | Cholestasis | 0.998 |
| 160921 | Bacterial Infections | 0.998 |
| 160921 | Immunologic Deficiency Syndromes | 0.998 |
| 161294 | Inflammation | 0.998 |
| 162350 | Epilepsy | 0.998 |
| 162350 | Neoplasm | 0.998 |
| 163040 | Parkinson Disease | 0.998 |
| 185914 | Immunologic Deficiency Syndromes | 0.998 |
| 185914 | Neoplasm | 0.998 |
| 222528 | Immunologic Deficiency Syndromes | 0.998 |
| 222528 | Thrombosis | 0.998 |
| 272944 | Immunologic Deficiency Syndromes | 0.998 |
| 358901 | Asthma | 0.998 |
| 358901 | Inflammation | 0.998 |
| 439246 | Immunologic Deficiency Syndromes | 0.998 |
| 439533 | Angina Pectoris | 0.998 |
| 439533 | Liver Cirrhosis | 0.998 |
| 439710 | Virus Diseases | 0.998 |
| 440832 | Renal Insufficiency, Chronic | 0.998 |
| 440832 | Thrombosis | 0.998 |
| 440967 | Virus Diseases | 0.998 |
| 441476 | Neoplasm | 0.998 |
| 441594 | Inflammation | 0.998 |
| 441663 | Neoplasm | 0.998 |
| 441737 | Cholestasis | 0.998 |
| 441970 | Thrombosis | 0.998 |
| 442408 | Immunologic Deficiency Syndromes | 0.998 |
| 442688 | Bacterial Infections | 0.998 |
| 442688 | Diabetes Mellitus | 0.998 |
| 443028 | Hypersensitivity | 0.998 |
| 443162 | Diarrhea | 0.998 |
| 443758 | Inflammation | 0.998 |
| 445858 | Hypersensitivity | 0.998 |
| 637542 | Angina Pectoris | 0.998 |
| 717531 | Virus Diseases | 0.998 |
| 1548910 | Thrombosis | 0.998 |
| 1549018 | Immunologic Deficiency Syndromes | 0.998 |
| 1549111 | Inflammation | 0.998 |
| 1549111 | Schizophrenia | 0.998 |
| 3081374 | Diabetes Mellitus | 0.998 |
| 3084288 | Bacterial Infections | 0.998 |
| 5280343 | Atherosclerosis | 0.998 |
| 5280343 | Renal Insufficiency, Chronic | 0.998 |
| 5280343 | Thrombosis | 0.998 |
| 5280378 | Angina Pectoris | 0.998 |
| 5280378 | Neoplasm | 0.998 |
| 5280794 | Bone Diseases, Developmental | 0.998 |
| 5280862 | Thrombosis | 0.998 |
| 5280933 | Fever | 0.998 |
| 5280934 | Angina Pectoris | 0.998 |
| 5281232 | Angina Pectoris | 0.998 |
| 5281252 | Bacterial Infections | 0.998 |
| 5281617 | Neoplasm | 0.998 |
| 5281635 | Atherosclerosis | 0.998 |
| 5281654 | Hyperlipidemias | 0.998 |
| 5281670 | Schizophrenia | 0.998 |
| 5281699 | Neoplasm | 0.998 |
| 5281779 | Fever | 0.998 |
| 5281817 | Immunologic Deficiency Syndromes | 0.998 |
| 5281855 | Liver Cirrhosis | 0.998 |
| 5281855 | Parkinson Disease | 0.998 |
| 5317357 | Immunologic Deficiency Syndromes | 0.998 |
| 5321018 | Neoplasm | 0.998 |
| 5322059 | Edema | 0.998 |
| 5458935 | Fever | 0.998 |
| 6439701 | Bacterial Infections | 0.998 |
| 6439701 | Edema | 0.998 |
| 6440205 | Schizophrenia | 0.998 |
| 6992244 | Hyperlipidemias | 0.998 |
| 10899740 | Renal Insufficiency, Chronic | 0.998 |
| 11600642 | Thrombosis | 0.998 |
| 11975273 | Angina Pectoris | 0.998 |
| 12442899 | Muscle Spasticity | 0.998 |
| 14849116 | Angina Pectoris | 0.998 |
| 20055073 | Neoplasm | 0.998 |
| 44257440 | Renal Insufficiency, Chronic | 0.998 |
| 46173994 | Immunologic Deficiency Syndromes | 0.998 |
| 54704420 | Diabetes Mellitus | 0.998 |
| 73817508 | Hypotension | 0.998 |
| 101659162 | Immunologic Deficiency Syndromes | 0.998 |
| 101937309 | Gout | 0.998 |
| 135438599 | Parkinson Disease | 0.998 |
| 125 | Asthma | 0.997 |
| 846 | Immunologic Deficiency Syndromes | 0.997 |
| 1183 | Gout | 0.997 |
| 3845 | Schizophrenia | 0.997 |
| 4680 | Gonadal Dysgenesis | 0.997 |
| 5154 | Osteoporosis | 0.997 |
| 6027 | Edema | 0.997 |
| 6549 | Epilepsy | 0.997 |
| 6989 | Inflammation | 0.997 |
| 7055 | Immunologic Deficiency Syndromes | 0.997 |
| 7428 | Edema | 0.997 |
| 7809 | Atherosclerosis | 0.997 |
| 8655 | Diabetes Mellitus | 0.997 |
| 8655 | Neoplasm | 0.997 |
| 10228 | Neoplasm | 0.997 |
| 10355 | Angina Pectoris | 0.997 |
| 10416 | Immunologic Deficiency Syndromes | 0.997 |
| 10795 | Cholestasis | 0.997 |
| 12038 | Inflammation | 0.997 |
| 16592 | Immunologic Deficiency Syndromes | 0.997 |
| 17100 | Diarrhea | 0.997 |
| 65057 | Inflammation | 0.997 |
| 72322 | Asthma | 0.997 |
| 72323 | Angina Pectoris | 0.997 |
| 72386 | Neoplasm | 0.997 |
| 72953 | Inflammation | 0.997 |
| 73160 | Parkinson Disease | 0.997 |
| 75142 | Neoplasm | 0.997 |
| 88944 | Renal Insufficiency, Chronic | 0.997 |
| 98914 | Diabetes Mellitus | 0.997 |
| 98914 | Immunologic Deficiency Syndromes | 0.997 |
| 124069 | Hyperlipidemias | 0.997 |
| 128735 | Neoplasm | 0.997 |
| 136330 | Thrombosis | 0.997 |
| 145742 | Stomach Ulcer | 0.997 |
| 155948 | Inflammation | 0.997 |
| 156336 | Inflammation | 0.997 |
| 156992 | Inflammation | 0.997 |
| 160511 | Inflammation | 0.997 |
| 160921 | Atherosclerosis | 0.997 |
| 162350 | Immunologic Deficiency Syndromes | 0.997 |
| 222284 | Hyperlipidemias | 0.997 |
| 222284 | Infertility, Female | 0.997 |
| 358901 | Angina Pectoris | 0.997 |
| 440832 | Inflammation | 0.997 |
| 441663 | Edema | 0.997 |
| 441737 | Epilepsy | 0.997 |
| 442408 | Fever | 0.997 |
| 442792 | Hypertension | 0.997 |
| 443758 | Atherosclerosis | 0.997 |
| 445639 | Immunologic Deficiency Syndromes | 0.997 |
| 637542 | Bacterial Infections | 0.997 |
| 643820 | Mycoses | 0.997 |
| 1585026 | Thrombosis | 0.997 |
| 3085014 | Edema | 0.997 |
| 5280343 | Neoplasm | 0.997 |
| 5280378 | Bacterial Infections | 0.997 |
| 5280378 | Cholestasis | 0.997 |
| 5280378 | Inflammation | 0.997 |
| 5280378 | Thrombosis | 0.997 |
| 5280537 | Neoplasm | 0.997 |
| 5280863 | Angina Pectoris | 0.997 |
| 5280933 | Renal Insufficiency, Chronic | 0.997 |
| 5281232 | Asthma | 0.997 |
| 5281232 | Neoplasm | 0.997 |
| 5281604 | Liver Cirrhosis | 0.997 |
| 5281670 | Angina Pectoris | 0.997 |
| 5281670 | Parkinson Disease | 0.997 |
| 5281703 | Rheumatic Diseases | 0.997 |
| 5281707 | Thrombosis | 0.997 |
| 5283316 | Gonadal Dysgenesis | 0.997 |
| 5283316 | Thrombosis | 0.997 |
| 5317321 | Inflammation | 0.997 |
| 5318889 | Bacterial Infections | 0.997 |
| 5320315 | Hyperlipidemias | 0.997 |
| 5320315 | Liver Cirrhosis | 0.997 |
| 5320351 | Atherosclerosis | 0.997 |
| 5320351 | Inflammation | 0.997 |
| 5321919 | Hypertension | 0.997 |
| 5321977 | Bacterial Infections | 0.997 |
| 5322059 | Renal Insufficiency, Chronic | 0.997 |
| 5322078 | Neoplasm | 0.997 |
| 5460291 | Diabetes Mellitus | 0.997 |
| 6371716 | Edema | 0.997 |
| 6429301 | Fever | 0.997 |
| 6439701 | Immunologic Deficiency Syndromes | 0.997 |
| 6442776 | Thrombosis | 0.997 |
| 9601691 | Neoplasm | 0.997 |
| 9889962 | Diabetes Mellitus | 0.997 |
| 9889962 | Liver Cirrhosis | 0.997 |
| 10899740 | Angina Pectoris | 0.997 |
| 10899740 | Menstruation Disturbances | 0.997 |
| 11062489 | Inflammation | 0.997 |
| 11250133 | Liver Cirrhosis | 0.997 |
| 12304519 | Hypotension | 0.997 |
| 12305360 | Bacterial Infections | 0.997 |
| 14656909 | Epilepsy | 0.997 |
| 14849116 | Inflammation | 0.997 |
| 16396350 | Edema | 0.997 |
| 20055073 | Menstruation Disturbances | 0.997 |
| 21117640 | Renal Insufficiency, Chronic | 0.997 |
| 21160900 | Fever | 0.997 |
| 46173956 | Nephritis | 0.997 |
| 56842002 | Edema | 0.997 |
| 101937309 | Angina Pectoris | 0.997 |
| 247 | Liver Cirrhosis | 0.996 |
| 342 | Edema | 0.996 |
| 750 | Diabetes Mellitus | 0.996 |
| 2758 | Cholestasis | 0.996 |
| 2758 | Neoplasm | 0.996 |
| 3218 | Mycoses | 0.996 |
| 3845 | Parkinson Disease | 0.996 |
| 6322 | Menstruation Disturbances | 0.996 |
| 6736 | Inflammation | 0.996 |
| 7013 | Thrombosis | 0.996 |
| 7428 | Inflammation | 0.996 |
| 7894 | Immunologic Deficiency Syndromes | 0.996 |
| 9015 | Epilepsy | 0.996 |
| 10207 | Inflammation | 0.996 |
| 11468 | Hypertension | 0.996 |
| 12038 | Mycoses | 0.996 |
| 12516 | Arrhythmias, Cardiac | 0.996 |
| 17100 | Bacterial Infections | 0.996 |
| 17100 | Inflammation | 0.996 |
| 32893 | Schizophrenia | 0.996 |
| 68313 | Neoplasm | 0.996 |
| 72322 | Angina Pectoris | 0.996 |
| 72322 | Diabetes Mellitus | 0.996 |
| 72344 | Nephritis | 0.996 |
| 72344 | Renal Insufficiency, Chronic | 0.996 |
| 72703 | Bacterial Infections | 0.996 |
| 72952 | Neoplasm | 0.996 |
| 73160 | Renal Insufficiency, Chronic | 0.996 |
| 79022 | Angina Pectoris | 0.996 |
| 82229 | Virus Diseases | 0.996 |
| 82755 | Inflammation | 0.996 |
| 92221 | Bacterial Infections | 0.996 |
| 96946 | Inflammation | 0.996 |
| 97214 | Inflammation | 0.996 |
| 98914 | Neoplasm | 0.996 |
| 99474 | Atherosclerosis | 0.996 |
| 119258 | Fever | 0.996 |
| 124069 | Bacterial Infections | 0.996 |
| 133590 | Edema | 0.996 |
| 133590 | Renal Insufficiency, Chronic | 0.996 |
| 146093 | Virus Diseases | 0.996 |
| 156051 | Parkinson Disease | 0.996 |
| 160237 | Immunologic Deficiency Syndromes | 0.996 |
| 160237 | Neoplasm | 0.996 |
| 160710 | Hyperlipidemias | 0.996 |
| 161294 | Atherosclerosis | 0.996 |
| 163040 | Osteoporosis | 0.996 |
| 167452 | Fever | 0.996 |
| 185914 | Hyperlipidemias | 0.996 |
| 222284 | Angina Pectoris | 0.996 |
| 272944 | Bacterial Infections | 0.996 |
| 276145 | Muscle Spasticity | 0.996 |
| 299645 | Immunologic Deficiency Syndromes | 0.996 |
| 439533 | Atherosclerosis | 0.996 |
| 439691 | Hypertension | 0.996 |
| 441476 | Bacterial Infections | 0.996 |
| 441737 | Fever | 0.996 |
| 442408 | Gonadal Dysgenesis | 0.996 |
| 445858 | Bacterial Infections | 0.996 |
| 479503 | Inflammation | 0.996 |
| 643820 | Hyperlipidemias | 0.996 |
| 1548910 | Atherosclerosis | 0.996 |
| 1548910 | Inflammation | 0.996 |
| 1548910 | Pneumonia | 0.996 |
| 1549018 | Inflammation | 0.996 |
| 1549025 | Atherosclerosis | 0.996 |
| 3080632 | Infertility, Female | 0.996 |
| 5280378 | Fever | 0.996 |
| 5280378 | Nephritis | 0.996 |
| 5280442 | Angina Pectoris | 0.996 |
| 5280442 | Hyperlipidemias | 0.996 |
| 5280863 | Inflammation | 0.996 |
| 5280863 | Neoplasm | 0.996 |
| 5280933 | Diabetes Mellitus | 0.996 |
| 5280933 | Parkinson Disease | 0.996 |
| 5280933 | Thrombosis | 0.996 |
| 5280934 | Diabetes Mellitus | 0.996 |
| 5281232 | Liver Cirrhosis | 0.996 |
| 5281325 | Diarrhea | 0.996 |
| 5281635 | Angina Pectoris | 0.996 |
| 5281670 | Inflammation | 0.996 |
| 5281779 | Gonadal Dysgenesis | 0.996 |
| 5283387 | Thrombosis | 0.996 |
| 5283640 | Hypertension | 0.996 |
| 5284421 | Angina Pectoris | 0.996 |
| 5312402 | Immunologic Deficiency Syndromes | 0.996 |
| 5317025 | Fever | 0.996 |
| 5318565 | Angina Pectoris | 0.996 |
| 5318565 | Neoplasm | 0.996 |
| 5320351 | Fever | 0.996 |
| 5320351 | Hypertension | 0.996 |
| 5320945 | Fever | 0.996 |
| 5321047 | Epilepsy | 0.996 |
| 5366074 | Fever | 0.996 |
| 5458935 | Cholestasis | 0.996 |
| 5488781 | Hyperlipidemias | 0.996 |
| 6433206 | Parkinson Disease | 0.996 |
| 6999980 | Thrombosis | 0.996 |
| 9841735 | Cholestasis | 0.996 |
| 10026486 | Immunologic Deficiency Syndromes | 0.996 |
| 11600642 | Schizophrenia | 0.996 |
| 12442899 | Hyperlipidemias | 0.996 |
| 14161394 | Angina Pectoris | 0.996 |
| 16132354 | Bacterial Infections | 0.996 |
| 21117640 | Nephritis | 0.996 |
| 56842002 | Immunologic Deficiency Syndromes | 0.996 |
| 73817508 | Bacterial Infections | 0.996 |
| 86736649 | Virus Diseases | 0.996 |
| 101615675 | Nephritis | 0.996 |
| 101659162 | Neoplasm | 0.996 |
| 101659163 | Neoplasm | 0.996 |
| 101659163 | Virus Diseases | 0.996 |
| 101937309 | Immunologic Deficiency Syndromes | 0.996 |
| 131674214 | Inflammation | 0.996 |
| 134767951 | Hypertension | 0.996 |
| 146158060 | Hyperlipidemias | 0.996 |
| 176 | Bacterial Infections | 0.995 |
| 311 | Inflammation | 0.995 |
| 338 | Immunologic Deficiency Syndromes | 0.995 |
| 460 | Neoplasm | 0.995 |
| 750 | Renal Insufficiency, Chronic | 0.995 |
| 1183 | Parkinson Disease | 0.995 |
| 2758 | Hypersensitivity | 0.995 |
| 2879 | Virus Diseases | 0.995 |
| 6199 | Neoplasm | 0.995 |
| 6448 | Neoplasm | 0.995 |
| 6549 | Bacterial Infections | 0.995 |
| 6549 | Hypertension | 0.995 |
| 6549 | Neoplasm | 0.995 |
| 6584 | Fever | 0.995 |
| 8434 | Cholestasis | 0.995 |
| 8655 | Inflammation | 0.995 |
| 9015 | Virus Diseases | 0.995 |
| 9064 | Inflammation | 0.995 |
| 9727 | Hypotension | 0.995 |
| 9727 | Inflammation | 0.995 |
| 10228 | Inflammation | 0.995 |
| 10355 | Atherosclerosis | 0.995 |
| 10795 | Hypertension | 0.995 |
| 11970 | Hypertension | 0.995 |
| 12127 | Neoplasm | 0.995 |
| 12575 | Fever | 0.995 |
| 12575 | Inflammation | 0.995 |
| 16666 | Neoplasm | 0.995 |
| 16724 | Immunologic Deficiency Syndromes | 0.995 |
| 16724 | Neoplasm | 0.995 |
| 19602 | Bacterial Infections | 0.995 |
| 22015 | Hyperlipidemias | 0.995 |
| 66948 | Bacterial Infections | 0.995 |
| 68081 | Neoplasm | 0.995 |
| 68313 | Diabetes Mellitus | 0.995 |
| 68352 | Asthma | 0.995 |
| 72322 | Neoplasm | 0.995 |
| 72322 | Pneumonia | 0.995 |
| 72386 | Virus Diseases | 0.995 |
| 72703 | Mycoses | 0.995 |
| 72953 | Neoplasm | 0.995 |
| 74840 | Immunologic Deficiency Syndromes | 0.995 |
| 88944 | Edema | 0.995 |
| 91458 | Osteoporosis | 0.995 |
| 91458 | Schizophrenia | 0.995 |
| 92221 | Hyperlipidemias | 0.995 |
| 94162 | Neoplasm | 0.995 |
| 100332 | Angina Pectoris | 0.995 |
| 100341 | Hypertension | 0.995 |
| 119268 | Bacterial Infections | 0.995 |
| 128735 | Schizophrenia | 0.995 |
| 128853 | Inflammation | 0.995 |
| 145858 | Hyperlipidemias | 0.995 |
| 145858 | Immunologic Deficiency Syndromes | 0.995 |
| 145937 | Inflammation | 0.995 |
| 155948 | Neoplasm | 0.995 |
| 156051 | Liver Cirrhosis | 0.995 |
| 157672 | Epilepsy | 0.995 |
| 160921 | Hypertension | 0.995 |
| 163040 | Immunologic Deficiency Syndromes | 0.995 |
| 168849 | Neoplasm | 0.995 |
| 272944 | Thrombosis | 0.995 |
| 334704 | Bacterial Infections | 0.995 |
| 334704 | Neoplasm | 0.995 |
| 354616 | Intestinal Diseases, Parasitic | 0.995 |
| 354735 | Inflammation | 0.995 |
| 439691 | Parkinson Disease | 0.995 |
| 440832 | Liver Cirrhosis | 0.995 |
| 440967 | Neoplasm | 0.995 |
| 440988 | Inflammation | 0.995 |
| 441970 | Fever | 0.995 |
| 442126 | Immunologic Deficiency Syndromes | 0.995 |
| 479503 | Immunologic Deficiency Syndromes | 0.995 |
| 637542 | Hemorrhage | 0.995 |
| 638072 | Hyperlipidemias | 0.995 |
| 717531 | Immunologic Deficiency Syndromes | 0.995 |
| 1548883 | Liver Cirrhosis | 0.995 |
| 1548910 | Asthma | 0.995 |
| 1548910 | Bacterial Infections | 0.995 |
| 1549111 | Epilepsy | 0.995 |
| 2724537 | Hyperlipidemias | 0.995 |
| 2734517 | Thrombosis | 0.995 |
| 3081374 | Inflammation | 0.995 |
| 3084331 | Mycoses | 0.995 |
| 5280343 | Diarrhea | 0.995 |
| 5280448 | Bacterial Infections | 0.995 |
| 5280489 | Infertility, Female | 0.995 |
| 5280544 | Bone Diseases, Developmental | 0.995 |
| 5280934 | Hyperlipidemias | 0.995 |
| 5280934 | Hypertension | 0.995 |
| 5280934 | Neoplasm | 0.995 |
| 5281331 | Virus Diseases | 0.995 |
| 5281617 | Immunologic Deficiency Syndromes | 0.995 |
| 5281635 | Inflammation | 0.995 |
| 5281654 | Hypertension | 0.995 |
| 5281703 | Parkinson Disease | 0.995 |
| 5282768 | Hypotension | 0.995 |
| 5283387 | Hypertension | 0.995 |
| 5320351 | Virus Diseases | 0.995 |
| 5321865 | Fever | 0.995 |
| 5372954 | Neoplasm | 0.995 |
| 5377686 | Atherosclerosis | 0.995 |
| 5491637 | Inflammation | 0.995 |
| 6440572 | Neoplasm | 0.995 |
| 6918700 | Diabetes Mellitus | 0.995 |
| 6918700 | Fever | 0.995 |
| 6999980 | Hypertension | 0.995 |
| 9841735 | Inflammation | 0.995 |
| 11600642 | Osteoporosis | 0.995 |
| 11600642 | Parkinson Disease | 0.995 |
| 11982272 | Schizophrenia | 0.995 |
| 12442899 | Virus Diseases | 0.995 |
| 21160900 | Arrhythmias, Cardiac | 0.995 |
| 46173956 | Edema | 0.995 |
| 54704420 | Angina Pectoris | 0.995 |
| 54704420 | Asthma | 0.995 |
| 54711004 | Immunologic Deficiency Syndromes | 0.995 |
| 73817508 | Hyperlipidemias | 0.995 |
| 101615675 | Atherosclerosis | 0.995 |
| 298 | Inflammation | 0.994 |
| 750 | Schizophrenia | 0.994 |
| 938 | Diabetes Mellitus | 0.994 |
| 938 | Epilepsy | 0.994 |
| 938 | Schizophrenia | 0.994 |
| 1183 | Neoplasm | 0.994 |
| 1183 | Schizophrenia | 0.994 |
| 2758 | Inflammation | 0.994 |
| 3314 | Bacterial Infections | 0.994 |
| 3314 | Epilepsy | 0.994 |
| 4680 | Neoplasm | 0.994 |
| 5610 | Immunologic Deficiency Syndromes | 0.994 |
| 6199 | Asthma | 0.994 |
| 6199 | Cough | 0.994 |
| 6549 | Fever | 0.994 |
| 6549 | Inflammation | 0.994 |
| 6549 | Liver Cirrhosis | 0.994 |
| 6584 | Virus Diseases | 0.994 |
| 7127 | Epilepsy | 0.994 |
| 7127 | Neoplasm | 0.994 |
| 7302 | Fever | 0.994 |
| 7809 | Angina Pectoris | 0.994 |
| 10355 | Schizophrenia | 0.994 |
| 10430 | Bone Diseases, Developmental | 0.994 |
| 14505 | Hyperlipidemias | 0.994 |
| 66948 | Hyperlipidemias | 0.994 |
| 72322 | Atherosclerosis | 0.994 |
| 72323 | Bacterial Infections | 0.994 |
| 73160 | Diabetes Mellitus | 0.994 |
| 73160 | Hyperlipidemias | 0.994 |
| 73160 | Inflammation | 0.994 |
| 73160 | Liver Cirrhosis | 0.994 |
| 76851 | Virus Diseases | 0.994 |
| 82755 | Angina Pectoris | 0.994 |
| 88944 | Inflammation | 0.994 |
| 97214 | Neoplasm | 0.994 |
| 99474 | Inflammation | 0.994 |
| 115247 | Inflammation | 0.994 |
| 155948 | Hypertension | 0.994 |
| 155948 | Immunologic Deficiency Syndromes | 0.994 |
| 160782 | Inflammation | 0.994 |
| 163040 | Inflammation | 0.994 |
| 167452 | Inflammation | 0.994 |
| 168849 | Inflammation | 0.994 |
| 185914 | Parkinson Disease | 0.994 |
| 188456 | Immunologic Deficiency Syndromes | 0.994 |
| 222528 | Angina Pectoris | 0.994 |
| 222528 | Atherosclerosis | 0.994 |
| 272944 | Inflammation | 0.994 |
| 299645 | Edema | 0.994 |
| 334704 | Atherosclerosis | 0.994 |
| 354616 | Diabetes Mellitus | 0.994 |
| 358901 | Bacterial Infections | 0.994 |
| 358901 | Pneumonia | 0.994 |
| 381152 | Intestinal Diseases, Parasitic | 0.994 |
| 439246 | Inflammation | 0.994 |
| 441737 | Arrhythmias, Cardiac | 0.994 |
| 442408 | Virus Diseases | 0.994 |
| 442437 | Immunologic Deficiency Syndromes | 0.994 |
| 442437 | Rheumatic Diseases | 0.994 |
| 442688 | Fever | 0.994 |
| 442885 | Liver Cirrhosis | 0.994 |
| 443162 | Hyperlipidemias | 0.994 |
| 443716 | Hypertension | 0.994 |
| 479503 | Neoplasm | 0.994 |
| 637511 | Hyperlipidemias | 0.994 |
| 641294 | Virus Diseases | 0.994 |
| 1548910 | Cough | 0.994 |
| 1548910 | Hyperlipidemias | 0.994 |
| 1549018 | Fever | 0.994 |
| 2724898 | Renal Insufficiency, Chronic | 0.994 |
| 2734517 | Gonadal Dysgenesis | 0.994 |
| 3085182 | Inflammation | 0.994 |
| 5280343 | Nephritis | 0.994 |
| 5280442 | Bacterial Infections | 0.994 |
| 5280448 | Angina Pectoris | 0.994 |
| 5280448 | Edema | 0.994 |
| 5280544 | Diabetes Mellitus | 0.994 |
| 5280794 | Bacterial Infections | 0.994 |
| 5280863 | Cholestasis | 0.994 |
| 5280863 | Thrombosis | 0.994 |
| 5280933 | Cholestasis | 0.994 |
| 5280933 | Epilepsy | 0.994 |
| 5280934 | Bacterial Infections | 0.994 |
| 5281232 | Inflammation | 0.994 |
| 5281404 | Edema | 0.994 |
| 5281604 | Renal Insufficiency, Chronic | 0.994 |
| 5281654 | Thrombosis | 0.994 |
| 5281670 | Liver Cirrhosis | 0.994 |
| 5281699 | Inflammation | 0.994 |
| 5281703 | Liver Cirrhosis | 0.994 |
| 5281817 | Thrombosis | 0.994 |
| 5281855 | Angina Pectoris | 0.994 |
| 5281855 | Neoplasm | 0.994 |
| 5284421 | Hypertension | 0.994 |
| 5317025 | Liver Cirrhosis | 0.994 |
| 5317238 | Hyperlipidemias | 0.994 |
| 5317357 | Thrombosis | 0.994 |
| 5317559 | Atherosclerosis | 0.994 |
| 5320351 | Angina Pectoris | 0.994 |
| 5321047 | Atherosclerosis | 0.994 |
| 5321047 | Virus Diseases | 0.994 |
| 5377686 | Virus Diseases | 0.994 |
| 5488781 | Inflammation | 0.994 |
| 6432173 | Immunologic Deficiency Syndromes | 0.994 |
| 6433206 | Neoplasm | 0.994 |
| 6440572 | Angina Pectoris | 0.994 |
| 6440572 | Atherosclerosis | 0.994 |
| 6442776 | Hypertension | 0.994 |
| 9889962 | Atherosclerosis | 0.994 |
| 10742927 | Immunologic Deficiency Syndromes | 0.994 |
| 10899740 | Arrhythmias, Cardiac | 0.994 |
| 11062489 | Diarrhea | 0.994 |
| 11062489 | Stomach Ulcer | 0.994 |
| 11975273 | Parkinson Disease | 0.994 |
| 11982272 | Inflammation | 0.994 |
| 54704420 | Immunologic Deficiency Syndromes | 0.994 |
| 73817507 | Cholestasis | 0.994 |
| 73817507 | Fever | 0.994 |
| 131675179 | Hyperlipidemias | 0.994 |
| 338 | Bacterial Infections | 0.993 |
| 750 | Hyperlipidemias | 0.993 |
| 1135 | Neoplasm | 0.993 |
| 1183 | Inflammation | 0.993 |
| 3218 | Liver Cirrhosis | 0.993 |
| 4133 | Mycoses | 0.993 |
| 4680 | Inflammation | 0.993 |
| 6054 | Bacterial Infections | 0.993 |
| 6549 | Immunologic Deficiency Syndromes | 0.993 |
| 6549 | Schizophrenia | 0.993 |
| 6736 | Cholestasis | 0.993 |
| 6736 | Fever | 0.993 |
| 6780 | Inflammation | 0.993 |
| 7428 | Renal Insufficiency, Chronic | 0.993 |
| 10102 | Inflammation | 0.993 |
| 10207 | Angina Pectoris | 0.993 |
| 10254 | Edema | 0.993 |
| 10393 | Immunologic Deficiency Syndromes | 0.993 |
| 10864 | Hyperlipidemias | 0.993 |
| 12575 | Cholestasis | 0.993 |
| 19009 | Bacterial Infections | 0.993 |
| 68081 | Inflammation | 0.993 |
| 68352 | Neoplasm | 0.993 |
| 69362 | Hypertension | 0.993 |
| 73160 | Thrombosis | 0.993 |
| 82755 | Atherosclerosis | 0.993 |
| 82755 | Edema | 0.993 |
| 82755 | Mycoses | 0.993 |
| 82755 | Renal Insufficiency, Chronic | 0.993 |
| 91458 | Inflammation | 0.993 |
| 91458 | Liver Cirrhosis | 0.993 |
| 96946 | Hyperlipidemias | 0.993 |
| 97679 | Intestinal Diseases, Parasitic | 0.993 |
| 97679 | Neoplasm | 0.993 |
| 98914 | Atherosclerosis | 0.993 |
| 99474 | Cough | 0.993 |
| 99474 | Neoplasm | 0.993 |
| 119258 | Inflammation | 0.993 |
| 119258 | Thrombosis | 0.993 |
| 119268 | Hyperlipidemias | 0.993 |
| 119831 | Inflammation | 0.993 |
| 124069 | Neoplasm | 0.993 |
| 155331 | Epilepsy | 0.993 |
| 160237 | Epilepsy | 0.993 |
| 160876 | Inflammation | 0.993 |
| 160876 | Neoplasm | 0.993 |
| 167452 | Thrombosis | 0.993 |
| 168849 | Virus Diseases | 0.993 |
| 174867 | Bone Diseases, Developmental | 0.993 |
| 185559 | Bacterial Infections | 0.993 |
| 191120 | Virus Diseases | 0.993 |
| 222284 | Asthma | 0.993 |
| 334704 | Liver Cirrhosis | 0.993 |
| 354616 | Gout | 0.993 |
| 354735 | Infertility, Female | 0.993 |
| 358901 | Cough | 0.993 |
| 381152 | Virus Diseases | 0.993 |
| 440832 | Nephritis | 0.993 |
| 441474 | Inflammation | 0.993 |
| 441594 | Intestinal Diseases, Parasitic | 0.993 |
| 441594 | Neoplasm | 0.993 |
| 441837 | Epilepsy | 0.993 |
| 442126 | Fever | 0.993 |
| 442437 | Inflammation | 0.993 |
| 442438 | Bacterial Infections | 0.993 |
| 442688 | Mycoses | 0.993 |
| 442792 | Gonadal Dysgenesis | 0.993 |
| 443028 | Thrombosis | 0.993 |
| 443156 | Intestinal Diseases, Parasitic | 0.993 |
| 479503 | Fever | 0.993 |
| 479503 | Thrombosis | 0.993 |
| 643820 | Bacterial Infections | 0.993 |
| 736186 | Thrombosis | 0.993 |
| 1548883 | Hyperlipidemias | 0.993 |
| 1548883 | Hypertension | 0.993 |
| 1549106 | Cholestasis | 0.993 |
| 1549106 | Fever | 0.993 |
| 3085014 | Renal Insufficiency, Chronic | 0.993 |
| 5280343 | Diabetes Mellitus | 0.993 |
| 5280343 | Fever | 0.993 |
| 5280343 | Liver Cirrhosis | 0.993 |
| 5280442 | Neoplasm | 0.993 |
| 5280445 | Schizophrenia | 0.993 |
| 5280448 | Neoplasm | 0.993 |
| 5280794 | Cholestasis | 0.993 |
| 5280794 | Hypersensitivity | 0.993 |
| 5280863 | Atherosclerosis | 0.993 |
| 5280863 | Hyperlipidemias | 0.993 |
| 5280933 | Atherosclerosis | 0.993 |
| 5281232 | Fever | 0.993 |
| 5281325 | Atherosclerosis | 0.993 |
| 5281604 | Inflammation | 0.993 |
| 5281617 | Thrombosis | 0.993 |
| 5281654 | Atherosclerosis | 0.993 |
| 5281699 | Bacterial Infections | 0.993 |
| 5282768 | Arrhythmias, Cardiac | 0.993 |
| 5317025 | Neoplasm | 0.993 |
| 5317357 | Inflammation | 0.993 |
| 5318042 | Immunologic Deficiency Syndromes | 0.993 |
| 5318889 | Fever | 0.993 |
| 5320351 | Intestinal Diseases, Parasitic | 0.993 |
| 5321018 | Thrombosis | 0.993 |
| 5321047 | Inflammation | 0.993 |
| 5321865 | Cholestasis | 0.993 |
| 5321865 | Inflammation | 0.993 |
| 5322059 | Thrombosis | 0.993 |
| 5366074 | Thrombosis | 0.993 |
| 5372954 | Diabetes Mellitus | 0.993 |
| 5460291 | Edema | 0.993 |
| 6440205 | Parkinson Disease | 0.993 |
| 6992244 | Bacterial Infections | 0.993 |
| 9841735 | Fever | 0.993 |
| 10899740 | Immunologic Deficiency Syndromes | 0.993 |
| 11051711 | Renal Insufficiency, Chronic | 0.993 |
| 11062489 | Fever | 0.993 |
| 11600642 | Epilepsy | 0.993 |
| 11600642 | Hyperlipidemias | 0.993 |
| 11600642 | Hypertension | 0.993 |
| 11600642 | Liver Cirrhosis | 0.993 |
| 11975273 | Atherosclerosis | 0.993 |
| 12304519 | Arrhythmias, Cardiac | 0.993 |
| 12304519 | Inflammation | 0.993 |
| 14161394 | Hyperlipidemias | 0.993 |
| 14849116 | Edema | 0.993 |
| 44257585 | Arrhythmias, Cardiac | 0.993 |
| 54704420 | Bacterial Infections | 0.993 |
| 71308174 | Diabetes Mellitus | 0.993 |
| 73817508 | Cholestasis | 0.993 |
| 101615675 | Edema | 0.993 |
| 101615675 | Renal Insufficiency, Chronic | 0.993 |
| 101921699 | Hyperlipidemias | 0.993 |
| 101937309 | Renal Insufficiency, Chronic | 0.993 |
| 135438599 | Atherosclerosis | 0.993 |
| 311 | Hemorrhage | 0.992 |
| 311 | Thrombosis | 0.992 |
| 2758 | Bacterial Infections | 0.992 |
| 2758 | Cough | 0.992 |
| 3845 | Inflammation | 0.992 |
| 5154 | Inflammation | 0.992 |
| 6448 | Hypertension | 0.992 |
| 7428 | Intestinal Diseases, Parasitic | 0.992 |
| 8554 | Thrombosis | 0.992 |
| 10931 | Inflammation | 0.992 |
| 12038 | Diabetes Mellitus | 0.992 |
| 12127 | Angina Pectoris | 0.992 |
| 13603 | Immunologic Deficiency Syndromes | 0.992 |
| 16666 | Immunologic Deficiency Syndromes | 0.992 |
| 16724 | Inflammation | 0.992 |
| 19009 | Mycoses | 0.992 |
| 72703 | Inflammation | 0.992 |
| 73160 | Atherosclerosis | 0.992 |
| 73160 | Bacterial Infections | 0.992 |
| 73160 | Fever | 0.992 |
| 73160 | Neoplasm | 0.992 |
| 73160 | Nephritis | 0.992 |
| 73160 | Virus Diseases | 0.992 |
| 75142 | Bacterial Infections | 0.992 |
| 82229 | Diarrhea | 0.992 |
| 82229 | Muscle Spasticity | 0.992 |
| 82229 | Mycoses | 0.992 |
| 89594 | Edema | 0.992 |
| 94162 | Virus Diseases | 0.992 |
| 97679 | Mycoses | 0.992 |
| 98914 | Inflammation | 0.992 |
| 99474 | Asthma | 0.992 |
| 99474 | Hyperlipidemias | 0.992 |
| 145937 | Neoplasm | 0.992 |
| 145937 | Parkinson Disease | 0.992 |
| 156051 | Hyperlipidemias | 0.992 |
| 156051 | Thrombosis | 0.992 |
| 160237 | Inflammation | 0.992 |
| 160237 | Liver Cirrhosis | 0.992 |
| 160492 | Schizophrenia | 0.992 |
| 160876 | Atherosclerosis | 0.992 |
| 162350 | Muscle Spasticity | 0.992 |
| 163040 | Schizophrenia | 0.992 |
| 169494 | Hypersensitivity | 0.992 |
| 185914 | Epilepsy | 0.992 |
| 222284 | Renal Insufficiency, Chronic | 0.992 |
| 354616 | Inflammation | 0.992 |
| 354616 | Schizophrenia | 0.992 |
| 358901 | Atherosclerosis | 0.992 |
| 439710 | Hyperlipidemias | 0.992 |
| 440966 | Edema | 0.992 |
| 440966 | Mycoses | 0.992 |
| 440967 | Mycoses | 0.992 |
| 442113 | Inflammation | 0.992 |
| 442688 | Neoplasm | 0.992 |
| 443156 | Bacterial Infections | 0.992 |
| 443158 | Schizophrenia | 0.992 |
| 443758 | Fever | 0.992 |
| 443758 | Parkinson Disease | 0.992 |
| 637511 | Diabetes Mellitus | 0.992 |
| 637542 | Atherosclerosis | 0.992 |
| 637542 | Neoplasm | 0.992 |
| 689075 | Neoplasm | 0.992 |
| 1548910 | Hypertension | 0.992 |
| 1548910 | Neoplasm | 0.992 |
| 1549025 | Angina Pectoris | 0.992 |
| 1549025 | Schizophrenia | 0.992 |
| 1742210 | Inflammation | 0.992 |
| 5280343 | Inflammation | 0.992 |
| 5280343 | Osteoporosis | 0.992 |
| 5280378 | Hyperlipidemias | 0.992 |
| 5280442 | Thrombosis | 0.992 |
| 5280445 | Inflammation | 0.992 |
| 5280448 | Hypertension | 0.992 |
| 5280448 | Inflammation | 0.992 |
| 5280544 | Hyperlipidemias | 0.992 |
| 5280544 | Immunologic Deficiency Syndromes | 0.992 |
| 5280794 | Gonadal Dysgenesis | 0.992 |
| 5280794 | Infertility, Female | 0.992 |
| 5280933 | Angina Pectoris | 0.992 |
| 5280934 | Atherosclerosis | 0.992 |
| 5281604 | Nephritis | 0.992 |
| 5281617 | Inflammation | 0.992 |
| 5281628 | Hyperlipidemias | 0.992 |
| 5281654 | Liver Cirrhosis | 0.992 |
| 5281699 | Hypotension | 0.992 |
| 5281703 | Bone Diseases, Developmental | 0.992 |
| 5281703 | Diabetes Mellitus | 0.992 |
| 5281707 | Gonadal Dysgenesis | 0.992 |
| 5281707 | Inflammation | 0.992 |
| 5281758 | Epilepsy | 0.992 |
| 5281817 | Inflammation | 0.992 |
| 5281855 | Inflammation | 0.992 |
| 5284371 | Muscle Spasticity | 0.992 |
| 5316141 | Bacterial Infections | 0.992 |
| 5316141 | Inflammation | 0.992 |
| 5317025 | Inflammation | 0.992 |
| 5317205 | Inflammation | 0.992 |
| 5317238 | Inflammation | 0.992 |
| 5320945 | Neoplasm | 0.992 |
| 5321919 | Inflammation | 0.992 |
| 5471349 | Hyperlipidemias | 0.992 |
| 6918700 | Cholestasis | 0.992 |
| 6918700 | Thrombosis | 0.992 |
| 12442899 | Immunologic Deficiency Syndromes | 0.992 |
| 16132354 | Inflammation | 0.992 |
| 21117640 | Edema | 0.992 |
| 44257585 | Bacterial Infections | 0.992 |
| 54704420 | Neoplasm | 0.992 |
| 54711004 | Cholestasis | 0.992 |
| 54711004 | Inflammation | 0.992 |
| 71306914 | Neoplasm | 0.992 |
| 73817508 | Arrhythmias, Cardiac | 0.992 |
| 73817508 | Stomach Ulcer | 0.992 |
| 101937309 | Rheumatic Diseases | 0.992 |
| 190 | Neoplasm | 0.991 |
| 247 | Fever | 0.991 |
| 247 | Inflammation | 0.991 |
| 311 | Fever | 0.991 |
| 753 | Cholestasis | 0.991 |
| 2758 | Asthma | 0.991 |
| 2758 | Pneumonia | 0.991 |
| 3218 | Immunologic Deficiency Syndromes | 0.991 |
| 3218 | Thrombosis | 0.991 |
| 4680 | Infertility, Female | 0.991 |
| 5154 | Asthma | 0.991 |
| 5154 | Neoplasm | 0.991 |
| 6199 | Inflammation | 0.991 |
| 6736 | Epilepsy | 0.991 |
| 7013 | Immunologic Deficiency Syndromes | 0.991 |
| 7428 | Neoplasm | 0.991 |
| 8655 | Bacterial Infections | 0.991 |
| 10228 | Liver Cirrhosis | 0.991 |
| 10228 | Schizophrenia | 0.991 |
| 11468 | Virus Diseases | 0.991 |
| 12575 | Immunologic Deficiency Syndromes | 0.991 |
| 17100 | Hyperlipidemias | 0.991 |
| 19009 | Virus Diseases | 0.991 |
| 68352 | Thrombosis | 0.991 |
| 72323 | Neoplasm | 0.991 |
| 72386 | Epilepsy | 0.991 |
| 72703 | Neoplasm | 0.991 |
| 72961 | Hyperlipidemias | 0.991 |
| 82755 | Virus Diseases | 0.991 |
| 88944 | Fever | 0.991 |
| 91458 | Asthma | 0.991 |
| 91458 | Neoplasm | 0.991 |
| 92221 | Neoplasm | 0.991 |
| 97214 | Immunologic Deficiency Syndromes | 0.991 |
| 99474 | Pneumonia | 0.991 |
| 119034 | Inflammation | 0.991 |
| 119034 | Renal Insufficiency, Chronic | 0.991 |
| 128853 | Immunologic Deficiency Syndromes | 0.991 |
| 145858 | Inflammation | 0.991 |
| 146093 | Asthma | 0.991 |
| 156336 | Immunologic Deficiency Syndromes | 0.991 |
| 157672 | Schizophrenia | 0.991 |
| 161294 | Hypertension | 0.991 |
| 185914 | Inflammation | 0.991 |
| 185914 | Pneumonia | 0.991 |
| 272944 | Epilepsy | 0.991 |
| 272944 | Fever | 0.991 |
| 354616 | Fever | 0.991 |
| 362908 | Edema | 0.991 |
| 439691 | Epilepsy | 0.991 |
| 441837 | Neoplasm | 0.991 |
| 442792 | Bacterial Infections | 0.991 |
| 442885 | Thrombosis | 0.991 |
| 444899 | Menstruation Disturbances | 0.991 |
| 444899 | Neoplasm | 0.991 |
| 445639 | Bacterial Infections | 0.991 |
| 445858 | Inflammation | 0.991 |
| 638072 | Angina Pectoris | 0.991 |
| 1274465 | Bacterial Infections | 0.991 |
| 2734517 | Infertility, Female | 0.991 |
| 3081374 | Angina Pectoris | 0.991 |
| 3081374 | Neoplasm | 0.991 |
| 5280343 | Bacterial Infections | 0.991 |
| 5280343 | Hyperlipidemias | 0.991 |
| 5280378 | Diabetes Mellitus | 0.991 |
| 5280442 | Diabetes Mellitus | 0.991 |
| 5280537 | Atherosclerosis | 0.991 |
| 5280544 | Neoplasm | 0.991 |
| 5280862 | Inflammation | 0.991 |
| 5280933 | Hyperlipidemias | 0.991 |
| 5280934 | Parkinson Disease | 0.991 |
| 5281232 | Cholestasis | 0.991 |
| 5281404 | Neoplasm | 0.991 |
| 5281654 | Schizophrenia | 0.991 |
| 5281658 | Inflammation | 0.991 |
| 5281703 | Thrombosis | 0.991 |
| 5281707 | Virus Diseases | 0.991 |
| 5281779 | Infertility, Female | 0.991 |
| 5316093 | Epilepsy | 0.991 |
| 5317559 | Bacterial Infections | 0.991 |
| 5318042 | Arrhythmias, Cardiac | 0.991 |
| 5318565 | Atherosclerosis | 0.991 |
| 5318889 | Liver Cirrhosis | 0.991 |
| 5320351 | Bacterial Infections | 0.991 |
| 5321919 | Bacterial Infections | 0.991 |
| 5321919 | Fever | 0.991 |
| 5322059 | Nephritis | 0.991 |
| 5322078 | Inflammation | 0.991 |
| 5354264 | Cholestasis | 0.991 |
| 5354264 | Fever | 0.991 |
| 5471349 | Inflammation | 0.991 |
| 6305177 | Cholestasis | 0.991 |
| 6999980 | Hypersensitivity | 0.991 |
| 11051711 | Edema | 0.991 |
| 11250133 | Atherosclerosis | 0.991 |
| 11600642 | Menstruation Disturbances | 0.991 |
| 11600642 | Neoplasm | 0.991 |
| 14161394 | Atherosclerosis | 0.991 |
| 14656909 | Bacterial Infections | 0.991 |
| 14849116 | Neoplasm | 0.991 |
| 16132354 | Intestinal Diseases, Parasitic | 0.991 |
| 21160900 | Hypotension | 0.991 |
| 21160900 | Inflammation | 0.991 |
| 44257440 | Edema | 0.991 |
| 44257585 | Stomach Ulcer | 0.991 |
| 46173956 | Renal Insufficiency, Chronic | 0.991 |
| 54711004 | Hyperlipidemias | 0.991 |
| 56842002 | Epilepsy | 0.991 |
| 73345262 | Neoplasm | 0.991 |
| 73817507 | Edema | 0.991 |
| 73817507 | Renal Insufficiency, Chronic | 0.991 |
| 146158953 | Immunologic Deficiency Syndromes | 0.991 |
| 5154 | Pneumonia | 0.990 |
| 6274 | Epilepsy | 0.990 |
| 6448 | Hyperlipidemias | 0.990 |
| 6549 | Renal Insufficiency, Chronic | 0.990 |
| 7041 | Epilepsy | 0.990 |
| 10207 | Atherosclerosis | 0.990 |
| 10207 | Hyperlipidemias | 0.990 |
| 12127 | Diarrhea | 0.990 |
| 16724 | Thrombosis | 0.990 |
| 17100 | Neoplasm | 0.990 |
| 22015 | Virus Diseases | 0.990 |
| 23752 | Fever | 0.990 |
| 72344 | Neoplasm | 0.990 |
| 73299 | Fever | 0.990 |
| 98914 | Angina Pectoris | 0.990 |
| 99474 | Angina Pectoris | 0.990 |
| 119034 | Parkinson Disease | 0.990 |
| 119268 | Immunologic Deficiency Syndromes | 0.990 |
| 119268 | Inflammation | 0.990 |
| 119831 | Epilepsy | 0.990 |
| 161294 | Hyperlipidemias | 0.990 |
| 222284 | Edema | 0.990 |
| 222284 | Pneumonia | 0.990 |
| 334704 | Angina Pectoris | 0.990 |
| 441837 | Inflammation | 0.990 |
| 442792 | Asthma | 0.990 |
| 443716 | Renal Insufficiency, Chronic | 0.990 |
| 457801 | Hyperlipidemias | 0.990 |
| 638072 | Parkinson Disease | 0.990 |
| 1742210 | Fever | 0.990 |
| 3000322 | Epilepsy | 0.990 |
| 5280442 | Mycoses | 0.990 |
| 5280489 | Bone Diseases, Developmental | 0.990 |
| 5280544 | Virus Diseases | 0.990 |
| 5280863 | Fever | 0.990 |
| 5281331 | Inflammation | 0.990 |
| 5281441 | Virus Diseases | 0.990 |
| 5281558 | Fever | 0.990 |
| 5281628 | Diabetes Mellitus | 0.990 |
| 5281628 | Epilepsy | 0.990 |
| 5281628 | Neoplasm | 0.990 |
| 5281635 | Liver Cirrhosis | 0.990 |
| 5281699 | Edema | 0.990 |
| 5281758 | Immunologic Deficiency Syndromes | 0.990 |
| 5281855 | Atherosclerosis | 0.990 |
| 5282713 | Bacterial Infections | 0.990 |
| 5317559 | Angina Pectoris | 0.990 |
| 5318889 | Inflammation | 0.990 |
| 5320315 | Inflammation | 0.990 |
| 5321047 | Schizophrenia | 0.990 |
| 5322059 | Menstruation Disturbances | 0.990 |
| 5322078 | Edema | 0.990 |
| 6992244 | Hypertension | 0.990 |
| 11170944 | Immunologic Deficiency Syndromes | 0.990 |
| 11600642 | Cholestasis | 0.990 |
| 11975273 | Immunologic Deficiency Syndromes | 0.990 |
| 12305360 | Virus Diseases | 0.990 |
| 16396350 | Virus Diseases | 0.990 |
| 17751030 | Hypertension | 0.990 |
| 71308174 | Hyperlipidemias | 0.990 |
| 101615675 | Angina Pectoris | 0.990 |
| 5280445 | Neoplasm | 0.989 |
| 5317205 | Intestinal Diseases, Parasitic | 0.989 |
| 7013 | Inflammation | 0.988 |
| 5284421 | Atherosclerosis | 0.988 |
| 10228 | Hypersensitivity | 0.987 |
| 439711 | Bacterial Infections | 0.987 |
| 443156 | Epilepsy | 0.987 |
| 712316 | Neoplasm | 0.987 |
| 119258 | Neoplasm | 0.986 |
| 185914 | Asthma | 0.986 |
| 5280863 | Liver Cirrhosis | 0.986 |
| 5320315 | Neoplasm | 0.986 |
| 6439701 | Liver Cirrhosis | 0.986 |
| 69362 | Cholestasis | 0.985 |
| 5281628 | Hypertension | 0.985 |
| 134767951 | Hypotension | 0.985 |
| 8655 | Schizophrenia | 0.984 |
| 99474 | Parkinson Disease | 0.984 |
| 6274 | Fever | 0.983 |
| 5321047 | Bacterial Infections | 0.983 |
| 5377686 | Bacterial Infections | 0.983 |
| 6199 | Pneumonia | 0.982 |
| 5281703 | Gout | 0.982 |
| 11600642 | Inflammation | 0.982 |
| 3845 | Neoplasm | 0.981 |
| 10207 | Neoplasm | 0.981 |
| 10355 | Neoplasm | 0.981 |
| 689075 | Diabetes Mellitus | 0.981 |
| 712316 | Immunologic Deficiency Syndromes | 0.981 |
| 66948 | Mycoses | 0.980 |
| 439533 | Bacterial Infections | 0.980 |
| 736186 | Inflammation | 0.980 |
| 5280862 | Fever | 0.980 |
| 5281707 | Hyperlipidemias | 0.980 |
| 5610 | Bacterial Infections | 0.978 |
| 6448 | Epilepsy | 0.978 |
| 8417 | Epilepsy | 0.978 |
| 443162 | Neoplasm | 0.978 |
| 2214 | Bone Diseases, Developmental | 0.977 |
| 7127 | Muscle Spasticity | 0.977 |
| 145858 | Angina Pectoris | 0.977 |
| 1549111 | Parkinson Disease | 0.977 |
| 7809 | Arrhythmias, Cardiac | 0.976 |
| 72323 | Inflammation | 0.976 |
| 5281331 | Diabetes Mellitus | 0.975 |
| 71306914 | Inflammation | 0.975 |
| 3218 | Neoplasm | 0.974 |
| 6274 | Atherosclerosis | 0.974 |
| 100341 | Neoplasm | 0.974 |
| 299645 | Virus Diseases | 0.974 |
| 5317025 | Bone Diseases, Developmental | 0.974 |
| 5366074 | Hyperlipidemias | 0.974 |
| 222284 | Atherosclerosis | 0.973 |
| 6989 | Neoplasm | 0.972 |
| 439533 | Epilepsy | 0.971 |
| 7041 | Hypotension | 0.970 |
| 1548910 | Angina Pectoris | 0.970 |
| 9750 | Edema | 0.969 |
| 586387 | Hypertension | 0.969 |
| 222528 | Hyperlipidemias | 0.968 |
| 11600642 | Fever | 0.968 |
| 222284 | Cholestasis | 0.967 |
| 5282713 | Edema | 0.967 |
| 72344 | Parkinson Disease | 0.966 |
| 89594 | Epilepsy | 0.966 |
| 5281441 | Hypertension | 0.966 |
| 5317321 | Neoplasm | 0.966 |
| 16132354 | Neoplasm | 0.966 |
| 68313 | Bacterial Infections | 0.965 |
| 72322 | Cough | 0.964 |
| 442126 | Neoplasm | 0.963 |
| 119268 | Neoplasm | 0.962 |
| 381152 | Neoplasm | 0.962 |
| 3085182 | Schizophrenia | 0.962 |
| 15693867 | Hyperlipidemias | 0.962 |
| 7809 | Hypotension | 0.961 |
| 68085 | Liver Cirrhosis | 0.961 |
| 3081374 | Atherosclerosis | 0.961 |
| 354735 | Gonadal Dysgenesis | 0.960 |
| 443162 | Inflammation | 0.960 |
| 689075 | Schizophrenia | 0.960 |
| 6371716 | Renal Insufficiency, Chronic | 0.960 |
| 2214 | Hypertension | 0.959 |
| 5317321 | Hypertension | 0.959 |
| 9727 | Arrhythmias, Cardiac | 0.958 |
| 9727 | Fever | 0.958 |
| 156051 | Atherosclerosis | 0.958 |
| 334704 | Inflammation | 0.958 |
| 5372954 | Parkinson Disease | 0.958 |
| 6992244 | Immunologic Deficiency Syndromes | 0.958 |
| 5321047 | Diabetes Mellitus | 0.957 |
| 6439701 | Hyperlipidemias | 0.956 |
| 124069 | Intestinal Diseases, Parasitic | 0.955 |
| 5282713 | Virus Diseases | 0.955 |
| 7469 | Virus Diseases | 0.954 |
| 3080635 | Virus Diseases | 0.954 |
| 5283316 | Hyperlipidemias | 0.954 |
| 6584 | Bacterial Infections | 0.952 |
| 5320945 | Inflammation | 0.952 |
| 16724 | Bacterial Infections | 0.951 |
| 86736649 | Hyperlipidemias | 0.951 |
| 445639 | Neoplasm | 0.950 |
| 3085182 | Parkinson Disease | 0.950 |
| 5281654 | Parkinson Disease | 0.950 |
| 11062489 | Cholestasis | 0.950 |
| 11982272 | Diabetes Mellitus | 0.950 |
| 298 | Epilepsy | 0.949 |
| 3080635 | Liver Cirrhosis | 0.949 |
| 5280343 | Hypertension | 0.949 |
| 5318042 | Epilepsy | 0.949 |
| 638072 | Neoplasm | 0.948 |
| 5281707 | Neoplasm | 0.948 |
| 338 | Inflammation | 0.947 |
| 354616 | Rheumatic Diseases | 0.947 |
| 5321047 | Neoplasm | 0.947 |
| 6989 | Virus Diseases | 0.946 |
| 156051 | Neoplasm | 0.946 |
| 162350 | Schizophrenia | 0.946 |
| 440966 | Hyperlipidemias | 0.946 |
| 6439701 | Neoplasm | 0.946 |
| 66948 | Cholestasis | 0.945 |
| 91458 | Parkinson Disease | 0.945 |
| 442885 | Hypertension | 0.945 |
| 3085182 | Epilepsy | 0.945 |
| 5318042 | Fever | 0.945 |
| 21160900 | Asthma | 0.945 |
| 712316 | Inflammation | 0.944 |
| 5312402 | Virus Diseases | 0.944 |
| 11250133 | Neoplasm | 0.944 |
| 1174 | Neoplasm | 0.943 |
| 9727 | Cholestasis | 0.943 |
| 133590 | Nephritis | 0.943 |
| 1742210 | Neoplasm | 0.942 |
| 9841735 | Diabetes Mellitus | 0.942 |
| 10254 | Immunologic Deficiency Syndromes | 0.941 |
| 22015 | Immunologic Deficiency Syndromes | 0.941 |
| 161294 | Bacterial Infections | 0.941 |
| 5281232 | Bacterial Infections | 0.941 |
| 17751030 | Immunologic Deficiency Syndromes | 0.941 |
| 354735 | Virus Diseases | 0.940 |
| 4680 | Arrhythmias, Cardiac | 0.939 |
| 10931 | Fever | 0.939 |
| 161294 | Angina Pectoris | 0.939 |
| 299645 | Bacterial Infections | 0.939 |
| 5280378 | Atherosclerosis | 0.939 |
| 5280442 | Inflammation | 0.939 |
| 2758 | Virus Diseases | 0.938 |
| 440966 | Bacterial Infections | 0.938 |
| 689075 | Parkinson Disease | 0.938 |
| 2724898 | Edema | 0.938 |
| 3080632 | Bacterial Infections | 0.938 |
| 6371716 | Nephritis | 0.938 |
| 54704420 | Atherosclerosis | 0.938 |
| 135438599 | Angina Pectoris | 0.938 |
| 457801 | Bacterial Infections | 0.936 |
| 586387 | Neoplasm | 0.936 |
| 717531 | Hyperlipidemias | 0.936 |
| 1549025 | Hypertension | 0.936 |
| 11250133 | Inflammation | 0.936 |
| 442437 | Gout | 0.935 |
| 5280445 | Diabetes Mellitus | 0.935 |
| 6549 | Asthma | 0.934 |
| 16928 | Angina Pectoris | 0.934 |
| 185914 | Cough | 0.934 |
| 2214 | Liver Cirrhosis | 0.933 |
| 5317238 | Cholestasis | 0.933 |
| 5320438 | Neoplasm | 0.933 |
| 100332 | Parkinson Disease | 0.932 |
| 73299 | Neoplasm | 0.931 |
| 188316 | Thrombosis | 0.931 |
| 10899740 | Atherosclerosis | 0.931 |
| 6918700 | Inflammation | 0.929 |
| 11982272 | Parkinson Disease | 0.929 |
| 54711004 | Fever | 0.929 |
| 6322 | Thrombosis | 0.928 |
| 7302 | Cholestasis | 0.928 |
| 155948 | Thrombosis | 0.928 |
| 174174 | Hypotension | 0.928 |
| 73817507 | Virus Diseases | 0.928 |
| 71306914 | Parkinson Disease | 0.927 |
| 311 | Cholestasis | 0.926 |
| 119034 | Intestinal Diseases, Parasitic | 0.926 |
| 5280933 | Inflammation | 0.926 |
| 5460291 | Hyperlipidemias | 0.926 |
| 101937309 | Atherosclerosis | 0.926 |
| 3845 | Epilepsy | 0.925 |
| 11468 | Inflammation | 0.924 |
| 161294 | Fever | 0.924 |
| 2758 | Fever | 0.922 |
| 5283387 | Bacterial Infections | 0.922 |
| 14849116 | Atherosclerosis | 0.922 |
| 5280489 | Gonadal Dysgenesis | 0.921 |
| 11600642 | Angina Pectoris | 0.921 |
| 237332 | Virus Diseases | 0.920 |
| 5282713 | Cholestasis | 0.920 |
| 5316093 | Edema | 0.920 |
| 160921 | Angina Pectoris | 0.919 |
| 222528 | Cholestasis | 0.919 |
| 71308174 | Atherosclerosis | 0.919 |
| 14340 | Virus Diseases | 0.918 |
| 442408 | Thrombosis | 0.918 |
| 54711004 | Virus Diseases | 0.918 |
| 750 | Nephritis | 0.917 |
| 442792 | Hyperlipidemias | 0.917 |
| 5281331 | Neoplasm | 0.917 |
| 443162 | Hypertension | 0.916 |
| 6440572 | Immunologic Deficiency Syndromes | 0.916 |
| 750 | Fever | 0.915 |
| 3080635 | Inflammation | 0.915 |
| 5280378 | Renal Insufficiency, Chronic | 0.914 |
| 5281670 | Atherosclerosis | 0.914 |
| 14161394 | Infertility, Female | 0.914 |
| 145858 | Edema | 0.913 |
| 441737 | Muscle Spasticity | 0.913 |
| 73817508 | Epilepsy | 0.913 |
| 69362 | Fever | 0.912 |
| 107217 | Virus Diseases | 0.911 |
| 185914 | Bacterial Infections | 0.911 |
| 5154 | Cough | 0.910 |
| 72323 | Atherosclerosis | 0.910 |
| 5281758 | Bacterial Infections | 0.910 |
| 443158 | Mycoses | 0.909 |
| 5280794 | Hyperlipidemias | 0.909 |
| 90351 | Bacterial Infections | 0.908 |
| 736186 | Diabetes Mellitus | 0.908 |
| 5280448 | Atherosclerosis | 0.908 |
| 6274 | Angina Pectoris | 0.907 |
| 1548883 | Cholestasis | 0.907 |
| 5318565 | Epilepsy | 0.907 |
| 11062489 | Bacterial Infections | 0.907 |
| 7302 | Bacterial Infections | 0.906 |
| 174174 | Atherosclerosis | 0.905 |
| 3084331 | Bacterial Infections | 0.905 |
| 6322 | Atherosclerosis | 0.904 |
| 88944 | Hypotension | 0.904 |
| 1548910 | Immunologic Deficiency Syndromes | 0.904 |
| 5284421 | Hyperlipidemias | 0.904 |
| 5321018 | Fever | 0.904 |
| 135438599 | Inflammation | 0.904 |
| 5281654 | Cholestasis | 0.903 |
| 439533 | Inflammation | 0.902 |
| 5322078 | Asthma | 0.902 |
| 188316 | Hypotension | 0.901 |
| 445639 | Virus Diseases | 0.901 |
| 637542 | Hyperlipidemias | 0.901 |
| 5281707 | Infertility, Female | 0.901 |
| 14656909 | Renal Insufficiency, Chronic | 0.887 |
| 442408 | Gout | 0.886 |
| 11250133 | Gonadal Dysgenesis | 0.886 |
| 717531 | Stomach Ulcer | 0.885 |
| 15693867 | Anemia | 0.885 |
| 5281654 | Hypersensitivity | 0.883 |
| 5318565 | Mycoses | 0.880 |
| 5322074 | Muscle Spasticity | 0.879 |
| 717531 | Anemia | 0.877 |
| 1135 | Renal Insufficiency, Chronic | 0.875 |
| 11600642 | Virus Diseases | 0.872 |
| 168849 | Stomach Ulcer | 0.871 |
| 6199 | Infertility, Female | 0.870 |
| 479503 | Hyperlipidemias | 0.866 |
| 5280445 | Hypotension | 0.866 |
| 5322078 | Fever | 0.866 |
| 1742210 | Osteoporosis | 0.865 |
| 146158060 | Neoplasm | 0.863 |
| 162350 | Diarrhea | 0.862 |
| 10393 | Mycoses | 0.861 |
| 21160900 | Infertility, Female | 0.846 |
| 5281817 | Liver Cirrhosis | 0.845 |
| 9018 | Thrombosis | 0.844 |
| 160492 | Hemorrhage | 0.844 |
| 9750 | Cough | 0.843 |
| 334704 | Thrombosis | 0.842 |
| 82755 | Gonadal Dysgenesis | 0.838 |
| 440832 | Bone Diseases, Developmental | 0.838 |
| 5281404 | Mycoses | 0.838 |
| 62065 | Stomach Ulcer | 0.837 |
| 717531 | Diabetes Mellitus | 0.835 |
| 12302744 | Diabetes Mellitus | 0.833 |
| 5281635 | Immunologic Deficiency Syndromes | 0.826 |
| 6199 | Gout | 0.823 |
| 21160900 | Bacterial Infections | 0.822 |
| 457801 | Asthma | 0.820 |
| 11600642 | Hypotension | 0.814 |
| 128735 | Liver Cirrhosis | 0.812 |
| 5281699 | Infertility, Female | 0.811 |
| 1713001 | Muscle Spasticity | 0.808 |
| 6432173 | Angina Pectoris | 0.808 |
| 1549106 | Hyperlipidemias | 0.807 |
| 13603 | Thrombosis | 0.805 |
| 736186 | Gonadal Dysgenesis | 0.804 |
| 76851 | Atherosclerosis | 0.803 |
| 443028 | Atherosclerosis | 0.800 |
| 5281855 | Rheumatic Diseases | 0.799 |
| 46173994 | Asthma | 0.798 |
| 71308174 | Infertility, Female | 0.798 |
| 72953 | Parkinson Disease | 0.797 |
| 5280933 | Virus Diseases | 0.795 |
| 5372954 | Atherosclerosis | 0.795 |
| 91458 | Fever | 0.794 |
| 31289 | Immunologic Deficiency Syndromes | 0.788 |
| 145742 | Nephritis | 0.788 |
| 5316957 | Hemorrhage | 0.788 |
| 5317238 | Infertility, Female | 0.785 |
| 5320945 | Intestinal Diseases, Parasitic | 0.785 |
| 299645 | Infertility, Female | 0.783 |
| 7150 | Diarrhea | 0.782 |
| 445638 | Arrhythmias, Cardiac | 0.781 |
| 145999601 | Bacterial Infections | 0.781 |
| 12575 | Hypotension | 0.780 |
| 5322074 | Epilepsy | 0.780 |
| 44258368 | Neoplasm | 0.780 |
| 16592 | Hypersensitivity | 0.775 |
| 3080632 | Asthma | 0.775 |
| 442388 | Bone Diseases, Developmental | 0.773 |
| 717531 | Bacterial Infections | 0.773 |
| 443028 | Gonadal Dysgenesis | 0.770 |
| 753 | Virus Diseases | 0.768 |
| 2758 | Hyperlipidemias | 0.768 |
| 6857370 | Virus Diseases | 0.765 |
| 124069 | Gonadal Dysgenesis | 0.756 |
| 442885 | Hypersensitivity | 0.756 |
| 443758 | Hypersensitivity | 0.756 |
| 16666 | Schizophrenia | 0.754 |
| 225936 | Hypersensitivity | 0.750 |
| 5281703 | Nephritis | 0.750 |
| 8299 | Gonadal Dysgenesis | 0.749 |
| 5320438 | Arrhythmias, Cardiac | 0.737 |
| 5317025 | Rheumatic Diseases | 0.735 |
| 12304519 | Hypertension | 0.734 |
| 5321018 | Immunologic Deficiency Syndromes | 0.731 |
| 68081 | Osteoporosis | 0.729 |
| 8434 | Liver Cirrhosis | 0.726 |
| 444899 | Liver Cirrhosis | 0.721 |
| 311 | Parkinson Disease | 0.713 |
| 5280448 | Asthma | 0.711 |
| 717531 | Diarrhea | 0.709 |
| 119831 | Neoplasm | 0.707 |
| 1548883 | Neoplasm | 0.704 |
| 335 | Muscle Spasticity | 0.696 |
| 174867 | Epilepsy | 0.690 |
| 3080635 | Diarrhea | 0.689 |
| 14161394 | Inflammation | 0.688 |
| 145999601 | Fever | 0.688 |
| 5280934 | Liver Cirrhosis | 0.686 |
| 12101 | Nephritis | 0.682 |
| 75142 | Hypotension | 0.682 |
| 1549018 | Liver Cirrhosis | 0.682 |
| 6971098 | Intestinal Diseases, Parasitic | 0.681 |
| 160237 | Hemorrhage | 0.676 |
| 4133 | Epilepsy | 0.673 |
| 10795 | Rheumatic Diseases | 0.672 |
| 11170944 | Menstruation Disturbances | 0.671 |
| 11970 | Inflammation | 0.668 |
| 5283640 | Muscle Spasticity | 0.667 |
| 5280343 | Cough | 0.666 |
| 128853 | Mycoses | 0.665 |
| 334704 | Hypertension | 0.665 |
| 6857370 | Cholestasis | 0.665 |
| 5280489 | Osteoporosis | 0.661 |
| 21117640 | Schizophrenia | 0.660 |
| 5320945 | Hemorrhage | 0.658 |
| 162350 | Intestinal Diseases, Parasitic | 0.655 |
| 442885 | Angina Pectoris | 0.655 |
| 6322 | Gout | 0.652 |
| 11982272 | Immunologic Deficiency Syndromes | 0.651 |
| 10742927 | Anemia | 0.650 |
| 10795 | Gonadal Dysgenesis | 0.645 |
| 5318042 | Nephritis | 0.644 |
| 11170944 | Diabetes Mellitus | 0.643 |
| 79035 | Intestinal Diseases, Parasitic | 0.641 |
| 5280863 | Pneumonia | 0.639 |
| 440967 | Fever | 0.637 |
| 21160900 | Gout | 0.637 |
| 17100 | Nephritis | 0.633 |
| 441476 | Infertility, Female | 0.630 |
| 460 | Virus Diseases | 0.629 |
| 643139 | Arrhythmias, Cardiac | 0.625 |
| 137947084 | Immunologic Deficiency Syndromes | 0.623 |
| 6184 | Infertility, Female | 0.616 |
| 17100 | Fever | 0.614 |
| 439691 | Diabetes Mellitus | 0.614 |
| 136330 | Epilepsy | 0.611 |
| 3314 | Renal Insufficiency, Chronic | 0.610 |
| 12575 | Edema | 0.609 |
| 3084288 | Epilepsy | 0.603 |
| 89594 | Pneumonia | 0.602 |
| 299645 | Arrhythmias, Cardiac | 0.600 |
| 5317238 | Parkinson Disease | 0.600 |
| 750 | Pneumonia | 0.599 |
| 72322 | Hypertension | 0.595 |
| 1549106 | Gonadal Dysgenesis | 0.595 |
| 156336 | Arrhythmias, Cardiac | 0.594 |
| 5281817 | Anemia | 0.594 |
| 6439701 | Schizophrenia | 0.594 |
| 20055073 | Epilepsy | 0.592 |
| 73299 | Hemorrhage | 0.591 |
| 5281699 | Intestinal Diseases, Parasitic | 0.588 |
| 16066 | Hypersensitivity | 0.586 |
| 311 | Stomach Ulcer | 0.585 |
| 1742210 | Intestinal Diseases, Parasitic | 0.585 |
| 8655 | Hyperlipidemias | 0.584 |
| 101921699 | Hypersensitivity | 0.583 |
| 247 | Infertility, Female | 0.581 |
| 5280343 | Muscle Spasticity | 0.581 |
| 101937309 | Diabetes Mellitus | 0.581 |
| 16066 | Nephritis | 0.577 |
| 222064 | Inflammation | 0.577 |
| 145999601 | Edema | 0.571 |
| 9750 | Fever | 0.570 |
| 5280442 | Edema | 0.569 |
| 338 | Bone Diseases, Developmental | 0.568 |
| 439711 | Renal Insufficiency, Chronic | 0.568 |
| 15693867 | Osteoporosis | 0.562 |
| 135438599 | Liver Cirrhosis | 0.560 |
| 10899740 | Diabetes Mellitus | 0.559 |
| 89594 | Bacterial Infections | 0.558 |
| 119058017 | Intestinal Diseases, Parasitic | 0.558 |
| 225936 | Epilepsy | 0.557 |
| 14505 | Immunologic Deficiency Syndromes | 0.551 |
| 134767951 | Gout | 0.550 |
| 667544 | Liver Cirrhosis | 0.549 |
| 445858 | Edema | 0.547 |
| 5488781 | Diabetes Mellitus | 0.545 |
| 10228 | Anemia | 0.544 |
| 5471349 | Infertility, Female | 0.544 |
| 190 | Liver Cirrhosis | 0.541 |
| 115247 | Cholestasis | 0.540 |
| 5471349 | Cough | 0.540 |
| 298 | Gout | 0.539 |
| 5317025 | Diabetes Mellitus | 0.536 |
| 14656909 | Osteoporosis | 0.534 |
| 443758 | Anemia | 0.533 |
| 17927 | Diabetes Mellitus | 0.532 |
| 2724537 | Arrhythmias, Cardiac | 0.530 |
| 5354264 | Mycoses | 0.530 |
| 68313 | Intestinal Diseases, Parasitic | 0.528 |
| 54711004 | Edema | 0.527 |
| 5320945 | Immunologic Deficiency Syndromes | 0.526 |
| 1549111 | Hypersensitivity | 0.525 |
| 7127 | Menstruation Disturbances | 0.520 |
| 96946 | Rheumatic Diseases | 0.520 |
| 8434 | Pneumonia | 0.519 |
| 163040 | Hypotension | 0.517 |
| 443758 | Rheumatic Diseases | 0.514 |
| 5280863 | Intestinal Diseases, Parasitic | 0.513 |
| 5280934 | Renal Insufficiency, Chronic | 0.511 |
| 439710 | Diarrhea | 0.510 |
| 8655 | Bone Diseases, Developmental | 0.507 |
| 9727 | Epilepsy | 0.505 |
| 188456 | Thrombosis | 0.505 |
| 3085014 | Gout | 0.503 |
| 5320315 | Intestinal Diseases, Parasitic | 0.503 |
| 6999980 | Hemorrhage | 0.502 |
| 90351 | Virus Diseases | 0.501 |
