# Supplementary material for: A Discovery Strategy for Active Compounds of Chinese Medicine Based on the Prediction Model of Compound-Disease Relationship
Source: J Oncol. 2022 Jul 8;2022:8704784. doi: 10.1155/2022/8704784 (PMC9286898; doi:10.1155/2022/8704784)
Supplement: Supplementary Materials — Table S1: prediction results of compounds of Chinese medicine. Table S2: importance score of antitumor compound features. Table S3: the network structure of the AlexNet model. Table S4: the network structure of the GoogLeNet model. Table S5: compounds of heat-clearing Chinese medicines in the SymMap database. [file 8704784.f1.zip › Table S2.docx]

Table S2: Importance score of anti-tumor compound features.

|  | Features | Importance score |
| --- | --- | --- |
| structure | Mi | 12.815 |
|  | RBF | 6.564 |
|  | BIC1 | 5.576 |
|  | MATS3v | 4.025 |
|  | SpMax1_Bh(m) | 3.755 |
|  | SpMAD_EA(dm) | 3.568 |
|  | SpMAD_AEA(dm) | 3.408 |
|  | Mor10u | 3.362 |
|  | Mor24e | 3.358 |
|  | Mor08s | 3.236 |
|  | P2v | 3.159 |
|  | HATS6m | 3.037 |
|  | HATSv | 3.026 |
|  | HATS1s | 3.014 |
|  | R4v+ | 2.940 |
|  | RTv+ | 2.936 |
|  | nCH2RX | 2.854 |
|  | H-047 | 2.847 |
| ADME | iLOGP | 9.419 |
|  | Fraction Csp3 | 7.440 |
|  | X Aromatic heavy atoms | 7.139 |
|  | Synthetic Accessibility | 6.686 |
|  | Consensus Log P | 6.156 |
|  | MLOGP | 6.016 |
|  | TPSA | 5.829 |
|  | MR | 5.799 |
|  | MW | 5.397 |
|  | Ali Log S | 5.380 |
|  | XLOGP3 | 5.306 |
|  | ESOL Log S | 5.282 |
|  | WLOGP | 5.074 |
