# Supplementary material for: A Discovery Strategy for Active Compounds of Chinese Medicine Based on the Prediction Model of Compound-Disease Relationship
Source: J Oncol. 2022 Jul 8;2022:8704784. doi: 10.1155/2022/8704784 (PMC9286898; doi:10.1155/2022/8704784)
Supplement: Supplementary Materials — Table S1: prediction results of compounds of Chinese medicine. Table S2: importance score of antitumor compound features. Table S3: the network structure of the AlexNet model. Table S4: the network structure of the GoogLeNet model. Table S5: compounds of heat-clearing Chinese medicines in the SymMap database. [file 8704784.f1.zip › Table S3.docx]

Table S3 The network structure of the AlexNet model

| order | Layers | size |
| --- | --- | --- |
| 1 | data | 227x227x1 |
| 2 | conv1 | 55x55x96 |
| 3 | relu1 | 55x55x96 |
| 4 | norm1 | 55x55x96 |
| 5 | pool1 | 27x27x96 |
| 6 | conv2 | 27x27x96 |
| 7 | relu2 | 27x27x96 |
| 8 | norm2 | 27x27x96 |
| 9 | pool2 | 13x13x256 |
| 10 | conv3 | 13x13x384 |
| 11 | relu3 | 13x13x384 |
| 12 | conv4 | 13x13x384 |
| 13 | relu4 | 13x13x384 |
| 14 | conv5 | 13x13x256 |
| 15 | relu5 | 13x13x256 |
| 16 | pool5 | 6x6x256 |
| 17 | fc6 | 1x1x4096 |
| 18 | relu6 | 1x1x4096 |
| 19 | drop6 | 1x1x4096 |
| 20 | fc7 | 1x1x4096 |
| 21 | relu7 | 1x1x4096 |
| 22 | drop7 | 1x1x4096 |
| 23 | fc8 | 1x1x1000 |
| 24 | prob | 1x1x1000 |
| 25 | output | - |
