# Supplementary material for: A Discovery Strategy for Active Compounds of Chinese Medicine Based on the Prediction Model of Compound-Disease Relationship
Source: J Oncol. 2022 Jul 8;2022:8704784. doi: 10.1155/2022/8704784 (PMC9286898; doi:10.1155/2022/8704784)
Supplement: Supplementary Materials — Table S1: prediction results of compounds of Chinese medicine. Table S2: importance score of antitumor compound features. Table S3: the network structure of the AlexNet model. Table S4: the network structure of the GoogLeNet model. Table S5: compounds of heat-clearing Chinese medicines in the SymMap database. [file 8704784.f1.zip › Table S4.docx]

Table S4 The network structure of the GoogLeNet model

| Order | Layer | Size |
| --- | --- | --- |
| 1 | data | 224x224x1 |
| 2 | conv1-7x7_s2 | 112x112x64 |
| 3 | conv1-relu_7x7 | 112x112x64 |
| 4 | pool1-3x3_s2 | 56x56x64 |
| 5 | pool1-norm1 | 56x56x64 |
| 6 | conv2-3x3_reduce | 56x56x64 |
| 7 | conv2-relu_3x3_reduce | 56x56x64 |
| 8 | conv2-3x3 | 56x56x192 |
| 9 | conv2-relu_3x3 | 56x56x192 |
| 10 | conv2-norm2 | 56x56x192 |
| 11 | pool2-3x3_s2 | 28x28x192 |
| 12 | inception_3a-5x5_reduce | 28x28x16 |
| 13 | inception_3a-3x3_reduce | 28x28x96 |
| 14 | inception_3a-relu_3x3_reduce | 28x28x96 |
| 15 | inception_3a-3x3 | 28x28x128 |
| 16 | inception_3a-relu_3x3 | 28x28x128 |
| 17 | inception_3a-1x1 | 28x28x64 |
| 18 | inception_3a-relu_1x1 | 28x28x64 |
| 19 | inception_3a-relu_5x5_reduce | 28x28x16 |
| 20 | inception_3a-5x5 | 28x28x32 |
| 21 | inception_3a-relu_5x5 | 28x28x32 |
| 22 | inception_3a-pool | 28x28x192 |
| 23 | inception_3a-pool_proj | 28x28x32 |
| 24 | inception_3a-relu_pool_proj | 28x28x32 |
| 25 | inception_3a-output | 28x28x256 |
| 26 | inception_3b-1x1 | 28x28x128 |
| 27 | inception_3b-pool | 28x28x256 |
| 28 | inception_3b-pool_proj | 28x28x64 |
| 29 | inception_3b-relu_pool_proj | 28x28x64 |
| 30 | inception_3b-relu_1x1 | 28x28x128 |
| 31 | inception_3b-5x5_reduce | 28x28x32 |
| 32 | inception_3b-relu_5x5_reduce | 28x28x32 |
| 33 | inception_3b-5x5 | 28x28x96 |
| 34 | inception_3b-3x3_reduce | 28x28x128 |
| 35 | inception_3b-relu_3x3_reduce | 28x28x128 |
| 36 | inception_3b-3x3 | 28x28x192 |
| 37 | inception_3b-relu_3x3 | 28x28x192 |
| 38 | inception_3b-relu_5x5 | 28x28x96 |
| 39 | inception_3b-output | 28x28x480 |
| 40 | pool3-3x3_s2 | 14x14x480 |
| 41 | inception_4a-3x3_reduce | 14x14x96 |
| 42 | inception_4a-relu_3x3_reduce | 14x14x96 |
| 43 | inception_4a-pool | 14x14x480 |
| 44 | inception_4a-5x5_reduce | 14x14x16 |
| 45 | inception_4a-relu_5x5_reduce | 14x14x16 |
| 46 | inception_4a-pool_proj | 14x14x64 |
| 47 | inception_4a-relu_pool_proj | 14x14x64 |
| 48 | inception_4a-1x1 | 14x14x192 |
| 49 | inception_4a-relu_1x1 | 14x14x192 |
| 50 | inception_4a-3x3 | 14x14x208 |
| 51 | inception_4a-relu_3x3 | 14x14x208 |
| 52 | inception_4a-5x5 | 14x14x48 |
| 53 | inception_4a-relu_5x5 | 14x14x48 |
| 54 | inception_4a-output | 14x14x512 |
| 55 | inception_4b-3x3_reduce | 14x14x112 |
| 56 | inception_4b-relu_3x3_reduce | 14x14x112 |
| 57 | inception_4b-pool | 14x14x512 |
| 58 | inception_4b-pool_proj | 14x14x64 |
| 59 | inception_4b-relu_pool_proj | 14x14x64 |
| 60 | inception_4b-5x5_reduce | 14x14x24 |
| 61 | inception_4b-relu_5x5_reduce | 14x14x24 |
| 62 | inception_4b-5x5 | 14x14x64 |
| 63 | inception_4b-relu_5x5 | 14x14x64 |
| 64 | inception_4b-1x1 | 14x14x160 |
| 65 | inception_4b-relu_1x1 | 14x14x160 |
| 66 | inception_4b-3x3 | 14x14x224 |
| 67 | inception_4b-relu_3x3 | 14x14x224 |
| 68 | inception_4b-output | 14x14x512 |
| 69 | inception_4c-5x5_reduce | 14x14x24 |
| 70 | inception_4c-pool | 14x14x512 |
| 71 | inception_4c-3x3_reduce | 14x14x128 |
| 72 | inception_4c-relu_3x3_reduce | 14x14x128 |
| 73 | inception_4c-pool_proj | 14x14x64 |
| 74 | inception_4c-relu_pool_proj | 14x14x64 |
| 75 | inception_4c-3x3 | 14x14x256 |
| 76 | inception_4c-relu_3x3 | 14x14x256 |
| 77 | inception_4c-1x1 | 14x14x128 |
| 78 | inception_4c-relu_1x1 | 14x14x128 |
| 79 | inception_4c-relu_5x5_reduce | 14x14x24 |
| 80 | inception_4c-5x5 | 14x14x64 |
| 81 | inception_4c-relu_5x5 | 14x14x64 |
| 82 | inception_4c-output | 14x14x512 |
| 83 | inception_4d-5x5_reduce | 14x14x32 |
| 84 | inception_4d-relu_5x5_reduce | 14x14x32 |
| 85 | inception_4d-1x1 | 14x14x112 |
| 86 | inception_4d-3x3_reduce | 14x14x144 |
| 87 | inception_4d-relu_1x1 | 14x14x112 |
| 88 | inception_4d-pool | 14x14x512 |
| 89 | inception_4d-relu_3x3_reduce | 14x14x144 |
| 90 | inception_4d-3x3 | 14x14x288 |
| 91 | inception_4d-relu_3x3 | 14x14x288 |
| 92 | inception_4d-pool_proj | 14x14x64 |
| 93 | inception_4d-relu_pool_proj | 14x14x64 |
| 94 | inception_4d-5x5 | 14x14x64 |
| 95 | inception_4d-relu_5x5 | 14x14x64 |
| 96 | inception_4d-output | 14x14x528 |
| 97 | inception_4e-5x5_reduce | 14x14x32 |
| 98 | inception_4e-3x3_reduce | 14x14x160 |
| 99 | inception_4e-relu_3x3_reduce | 14x14x160 |
| 100 | inception_4e-3x3 | 14x14x320 |
| 101 | inception_4e-relu_3x3 | 14x14x320 |
| 102 | inception_4e-relu_5x5_reduce | 14x14x32 |
| 103 | inception_4e-5x5 | 14x14x128 |
| 104 | inception_4e-relu_5x5 | 14x14x128 |
| 105 | inception_4e-pool | 14x14x528 |
| 106 | inception_4e-pool_proj | 14x14x128 |
| 107 | inception_4e-relu_pool_proj | 14x14x128 |
| 108 | inception_4e-1x1 | 14x14x256 |
| 109 | inception_4e-relu_1x1 | 14x14x256 |
| 110 | inception_4e-output | 14x14x832 |
| 111 | pool4-3x3_s2 | 7x7x832 |
| 112 | inception_5a-3x3_reduce | 7x7x160 |
| 113 | inception_5a-5x5_reduce | 7x7x32 |
| 114 | inception_5a-pool | 7x7x832 |
| 115 | inception_5a-1x1 | 7x7x256 |
| 116 | inception_5a-relu_5x5_reduce | 7x7x32 |
| 117 | inception_5a-5x5 | 7x7x128 |
| 118 | inception_5a-relu_3x3_reduce | 7x7x160 |
| 119 | inception_5a-3x3 | 7x7x320 |
| 120 | inception_5a-relu_3x3 | 7x7x320 |
| 121 | inception_5a-relu_5x5 | 7x7x128 |
| 122 | inception_5a-relu_1x1 | 7x7x256 |
| 123 | inception_5a-pool_proj | 7x7x128 |
| 124 | inception_5a-relu_pool_proj | 7x7x128 |
| 125 | inception_5a-output | 7x7x832 |
| 126 | inception_5b-1x1 | 7x7x384 |
| 127 | inception_5b-pool | 7x7x832 |
| 128 | inception_5b-relu_1x1 | 7x7x384 |
| 129 | inception_5b-5x5_reduce | 7x7x48 |
| 130 | inception_5b-relu_5x5_reduce | 7x7x48 |
| 131 | inception_5b-5x5 | 7x7x128 |
| 132 | inception_5b-relu_5x5 | 7x7x128 |
| 133 | inception_5b-pool_proj | 7x7x128 |
| 134 | inception_5b-relu_pool_proj | 7x7x128 |
| 135 | inception_5b-3x3_reduce | 7x7x192 |
| 136 | inception_5b-relu_3x3_reduce | 7x7x192 |
| 137 | inception_5b-3x3 | 7x7x384 |
| 138 | inception_5b-relu_3x3 | 7x7x384 |
| 139 | inception_5b-output | 7x7x1024 |
| 140 | pool5-7x7_s1 | 7x7x1024 |
| 141 | pool5-drop_7x7_s1 | 7x7x1024 |
| 142 | fc | 1x1x2 |
| 143 | prob | 1x1x2 |
| 144 | classoutput | - |
