# Supplementary material for: A Discovery Strategy for Active Compounds of Chinese Medicine Based on the Prediction Model of Compound-Disease Relationship
Source: J Oncol. 2022 Jul 8;2022:8704784. doi: 10.1155/2022/8704784 (PMC9286898; doi:10.1155/2022/8704784)
Supplement: Supplementary Materials — Table S1: prediction results of compounds of Chinese medicine. Table S2: importance score of antitumor compound features. Table S3: the network structure of the AlexNet model. Table S4: the network structure of the GoogLeNet model. Table S5: compounds of heat-clearing Chinese medicines in the SymMap database. [file 8704784.f1.zip › Table S5.docx]

Table S5 Compounds of heat-clearing Chinese medicines in the SymMap database

| Molecule_name | OB_score | CAS_id | PubChem_CID |
| --- | --- | --- | --- |
| 4-Hydroxybenzyl Alcohol | 55.186 | 623-05-2 | 125 |
| Acetic Acid | 47.865 | 110-49-6\|64-19-7 | 176 |
| Adenine | 62.806 | 73-24-5\|73-42-5 | 190 |
| Betaine | 40.922 | 107-43-7 | 247 |
| Cam | 67.174 | 76-22-2 | 298 |
| Citric Acid | 56.220 | 77-92-9 | 311 |
| O-Cresol | 62.454 | 95-48-7 | 335 |
| Salicylic Acid | 32.127 | 69-72-7 | 338 |
| Crs | 53.955 | 108-39-4 | 342 |
| Guasol | 51.595 | 8021-39-4 | 460 |
| Otan | 58.602 | 96-26-4 | 670 |
| Gly | 48.737 | 32817-15-5 | 750 |
| Ifp | 72.874 | 8043-29-6 | 753 |
| Vanillic Acid | 35.472 | 121-34-6 | 846 |
| Nicotinic Acid | 47.645 | 123574-58-3\|59-67-6 | 938 |
| Thy | 74.198 | 65-71-4 | 1135 |
| Toluene | 42.580 | 108-88-3 | 1140 |
| Uracil | 42.526 | 66-22-8 | 1174 |
| Vanillin | 51.996 | 121-33-5 | 1183 |
| Apocynin | 31.712 | 498-02-2 | 2214 |
| 1,8-Cineole | 39.729 | 470-82-6 | 2758 |
| Pcr | 51.986 | 72269-62-6 | 2879 |
| Embelin | 37.718 | 550-24-3 | 3218 |
| Eugenol | 56.242 | 97-53-0 | 3314 |
| Kyna | 44.716 | 13593-94-7 | 3845 |
| Methyl Salicylate | 42.550 | 119-36-8 | 4133 |
| Quinone | 33.579 | 19052-63-2 | 4650 |
| Papaverine | 64.043 | 58-74-2 | 4680 |
| Sanguinarine | 37.808 | 2447-54-3 | 5154 |
| Tyramine | 45.109 | 51-67-2 | 5610 |
| Xys | 58.742 | 6763-34-4 | 6027 |
| Pel | 44.034 | 60-12-8 | 6054 |
| Hexanal | 55.707 | 66-25-1 | 6184 |
| Psoralen | 33.061 | 66-97-7 | 6199 |
| Istidina | 53.175 | 30641-68-0 | 6274 |
| L- | 47.642 | 142-49-4 | 6322 |
| L-Bornyl Acetate | 65.521 | 6626-35-3 | 6448 |
| Izoforon | 44.983 | 78-59-1 | 6544 |
| Linalool | 49.367 | 78-70-6 | 6549 |
| Tereton | 40.172 | 79-20-9 | 6584 |
| Skatol | 69.669 | 83-34-1 | 6736 |
| Morkit | 56.101 | 84-65-1 | 6780 |
| Thymol | 41.474 | 89-83-8 | 6989 |
| Pana | 50.347 | 219315-45-4 | 7013 |
| Aldrich | 37.496 | 91-10-1 | 7041 |
| 2-Methylnaphthalene | 33.685 | 7419-61-6\|91-57-6 | 7055 |
| Methyleugenol | 73.360 | 6380-24-1\|93-15-2 | 7127 |
| Clorius | 45.990 | 93-58-3 | 7150 |
| Gbl | 76.906 | 187997-16-6 | 7302 |
| Methylgallate | 30.908 | 99-24-1 | 7428 |
| Piceol | 36.804 | 99-93-4 | 7469 |
| P-Xylene | 48.743 | 106-42-3 | 7809 |
| Len | 62.940 | 107-85-7 | 7894 |
| Nonanoic Acid | 40.509 | 112-05-0 | 8158 |
| Acetol | 53.934 | 116-09-6 | 8299 |
| Scoparone | 74.755 | 120-08-1 | 8417 |
| Sobrol A | 64.982 | 120-47-8 | 8434 |
| Mipax | 57.397 | 131-11-3 | 8554 |
| Syringaldehyde | 67.061 | 134-96-3 | 8655 |
| Nonanol | 33.194 | 143-08-8 | 8914 |
| Mehq | 43.981 | 150-76-5 | 9015 |
| Phytol | 33.824 | 150-86-7\|7541-49-3 | 9018 |
| (+)-Catechin | 54.826 | 154-23-4 | 9064 |
| N-Methyltyramine | 75.518 | 370-98-9 | 9727 |
| Cir | 52.960 | 372-75-8 | 9750 |
| Clovene | 46.494 | 469-92-1 | 10102 |
| Aloe-Emodin | 83.380 | 481-72-1 | 10207 |
| Osthol | 38.751 | 484-12-8 | 10228 |
| Mesitaldehyde | 37.803 | 487-68-3 | 10254 |
| Coumaran | 50.896 | 496-16-2 | 10329 |
| Arecaine | 84.343 | 499-04-7 | 10355 |
| Tyrosol | 33.812 | 501-94-0 | 10393 |
| Malvic Acid | 30.988 | 503-05-9 | 10416 |
| Iva | 62.168 | 92634-50-9 | 10430 |
| Methyl Formyl | 30.340 | 534-15-6 | 10795 |
| Amylbenzene | 34.341 | 27458-20-4 | 10864 |
| Senecic Acid | 62.325 | 541-47-9 | 10931 |
| Methyl Propylate | 69.053 | 554-12-1 | 11124 |
| 1-Terpineol | 49.834 | 586-82-3 | 11468 |
| Ohp | 33.653 | 614-75-5 | 11970 |
| Ethyl Vanillate | 31.111 | 617-05-0 | 12038 |
| M-Ethylphenol | 51.303 | 620-17-7 | 12101 |
| Isovanillin | 31.006 | 621-59-0 | 12127 |
| Ethyl Senecioate | 30.731 | 638-10-8 | 12516 |
| Isovanillic Acid | 39.424 | 645-08-9 | 12575 |
| N-Methylvalerolactam | 82.452 | 931-20-4 | 13603 |
| Hydroferulic | 34.674 | 1135-23-5 | 14340 |
| Acetylfuran | 49.633 | 80145-44-4 | 14505 |
| Dibutyl Terephthalate | 62.991 | 1962-75-0 | 16066 |
| 2,3-Dithiahexane | 70.144 | 2179-60-4 | 16592 |
| Menthol | 43.312 | 1490-04-6\|89-78-1\|98167-53-4 | 16666 |
| (S)-Carvone | 47.426 | 53763-73-8 | 16724 |
| Homovanillyl Alcohol | 38.200 | 2380-78-1 | 16928 |
| Alpha-Terpineol | 34.009 | 98-55-5 | 17100 |
| 6-Ethylresorcinol | 46.448 | 2896-60-8 | 17927 |
| Palmatine | 64.601 | 3486-67-7 | 19009 |
| Pentylfuran | 54.588 | 64079-01-2 | 19602 |
| 3,3-Dimethylbutylamine | 56.608 | 15673-00-4 | 19709 |
| Isoxylaldehyde | 38.850 | 5779-94-2 | 22015 |
| Tetramethylsuccinamide | 68.786 | 7334-51-2 | 23752 |
| Nonanal | 40.276 | 75718-12-6 | 31289 |
| Butylated Hydroxytoluene | 40.020 | 128-37-0\|50356-19-9 | 31404 |
| Levamfetamine | 44.320 | 156-34-3 | 32893 |
| Dob | 88.184 | 89-86-1 | 62065 |
| Sudan Iii | 84.066 | 85-86-9 | 62331 |
| Diphencyprone | 103.255 | 886-38-4 | 65057 |
| Protoanemonin | 44.068 | 108-28-1 | 66948 |
| Isoimperatorin | 45.464 | 482-45-1 | 68081 |
| Dictamine | 31.389 | 484-29-7 | 68085 |
| Hordenine | 46.957 | 539-15-1 | 68313 |
| Tulipane | 75.161 | 547-65-9 | 68352 |
| Butyric Acid, 3-Hydroxy-3-Methyl- | 31.905 | 88221-74-3 | 69362 |
| (R)-Ornithine | 90.502 | 410523-47-6 | 71082 |
| Coptisine | 30.672 | 3486-66-6 | 72322 |
| Jatrorrhizine | 30.437 | 3621-38-3 | 72323 |
| Nobiletin | 61.669 | 10236-47-2\|478-01-3 | 72344 |
| Thiarubrine A | 71.025 | 63543-09-9 | 72386 |
| Berberrubine | 35.736 | 15401-69-1 | 72703 |
| Yadanzioside B | 46.162 | 95258-18-7 | 72952 |
| Bruceoside B | 56.543 | 69687-69-0 | 72953 |
| Yadanzioside M | 45.041 | 101559-99-3 | 72961 |
| (-)-Catechin | 49.676 | 154-23-4 | 73160 |
| Hederagenin | 36.914 | 465-99-6 | 73299 |
| Z-Leu-Oh | 113.778 | 61-90-5 | 74840 |
| Beta-Hydroxypropiovanillone | 34.954 | 2196-18-1 | 75142 |
| 4-Formyl-2,6-Xylenol | 52.896 | 2233-18-3 | 75222 |
| P-Allyltoluene | 36.405 | 3333-13-9 | 76851 |
| Cyclofenchene | 42.269 | 488-97-1 | 79022 |
| Tricyclene | 36.113 | 508-32-7 | 79035 |
| Delta-Terpineol | 55.110 | 7299-42-5 | 81722 |
| L-Alpha-Fenchone | 72.639 | 7787-20-4 | 82229 |
| Hydroxytyrosol | 57.569 | 10597-60-1 | 82755 |
| 3,5-Dimethyl-P-Anisic Acid | 61.105 | 21553-46-8 | 88944 |
| Nicotine | 77.668 | 1954/11/5\|54-11-5 | 89594 |
| Acoradiene | 36.726 | 24048-44-0 | 90351 |
| Aucubin | 35.560 | 479-98-1 | 91458 |
| D-Camphene | 34.979 | 5794-03-6 | 92221 |
| Sugiol | 36.114 | 511-05-7 | 94162 |
| Lobelanidine | 60.530 | 552-72-7 | 96946 |
| Eupatorin | 30.232 | 855-96-9 | 97214 |
| Norsanguinarine | 45.039 | 522-30-5 | 97679 |
| Arnebin 7 | 73.848 | 43043-74-9 | 98914 |
| Diosgenin | 80.878 | 512-04-9 | 99474 |
| Loliolide | 44.662 | 11028-27-6 | 100332 |
| Griffonilide | 43.152 | 61371-55-9 | 100341 |
| Methyl 2,4-Dihydroxyphenylacetate | 34.710 | 67828-42-6 | 105727 |
| Fenchylacetate | 109.323 | 13851-11-1 | 107217 |
| Penniclavin | 48.155 | 519-13-1 | 115247 |
| Asiatic Acid | 41.383 | 464-92-6 | 119034 |
| Astilbin | 36.462 | 29838-67-3 | 119258 |
| Phaseollidin | 52.043 | 37831-70-2 | 119268 |
| Cedrenol | 108.563 | 28231-03-0 | 119831 |
| Vaccenic Acid | 33.128 | 693-72-1 | 122325 |
| Dihydrosanguinarine | 59.313 | 3606-45-9 | 124069 |
| 2H-3,9A-Methano-1-Benzoxepin-9-Methanol, Octahydro-2,2,5A-Trimethyl-, (3R-(3Alpha,5Aalpha,9Alpha,9Aalpha))- | 98.378 | 105013-72-7 | 128735 |
| Delphinidin | 40.635 | 528-53-0 | 128853 |
| Ea-Fructofuranoside | 47.327 | 81024-99-9 | 133590 |
| Eucarvone | 53.136 | 503-93-5 | 136330 |
| Prolinum | 77.575 | 18875-45-1 | 145742 |
| Anthocyanin | 55.346 | 11029-12-2 | 145858 |
| Eckol | 87.060 | 88798-74-7 | 145937 |
| Gossypetin Hexamethyl Ether | 42.843 | 7741-47-1 | 146093 |
| Stemonine | 81.747 | 20460-41-7 | 155331 |
| Atractylenolide Iii | 68.113 | 73030-71-4 | 155948 |
| Silymonin | 81.814 | 70815-31-5 | 156051 |
| Decussine | 39.830 | 75375-52-9 | 156336 |
| 5,8,2'-Trihydroxy-7-Methoxyflavone | 37.008 | 77056-20-3 | 156992 |
| D-Nicotine | 76.466 | 1596-94-7 | 157672 |
| Cirsiliol | 43.462 | 34334-69-5 | 160237 |
| Guvacoline | 32.671 | 495-19-2 | 160492 |
| Abrine | 70.832 | 526-31-8 | 160511 |
| Cnidilide | 77.551 | 3674-03-1 | 160710 |
| 4-Ethoxycarbonyl-2-Quinolone | 66.968 | 5466-27-3 | 160782 |
| Epiberberine | 43.092 | 6873/9/2 | 160876 |
| Ethoxychelerythrine | 62.213 | 79559-55-0 | 160921 |
| Peimisine | 57.402 | 19773-24-1 | 161294 |
| Isovitexin | 31.295 | 38953-85-4\|61838-34-4 | 162350 |
| Aucubigenin | 101.770 | 64274-28-8 | 163040 |
| Diayangambin | 63.840 | 21453-68-9 | 167452 |
| Pectolinarin | 47.616 | 28978-02-1 | 168849 |
| Arteglasin A | 52.449 | 33204-39-6 | 169494 |
| Atropine | 34.528 | 51-55-8 | 174174 |
| Azuleno(4,5-B)Furan-2(3H)-One, 4-(Acetyloxy)-3A,4,5,6,6A,7,9A,9B-Octahydro-6-Hydroxy-6,9-Dimethyl-3-Methylene-, (3Ar-(3Aalpha,4Alpha,6Alpha,6Aalpha,9Aalpha,9Bbeta))- | 68.437 | 20482-33-1 | 174867 |
| 24240-05-9 | 53.750 | 24240-05-9 | 185559 |
| Dihydroresveratrol | 87.273 | 58436-28-5 | 185914 |
| Moslosooflavone | 44.088 | 3570-62-5 | 188316 |
| Ineketone | 37.145 | 62574-18-9 | 188456 |
| Erythrocentaurin | 42.806 | 50276-98-7 | 191120 |
| 2,2-Dimethylchroman-6-Carboxylic Acid | 52.478 | 2039-47-6 | 222064 |
| Beta-Sitosterol | 36.914 | 83-46-5 | 222284 |
| Deoxycholic Acid | 40.723 | 83-44-3 | 222528 |
| Bu3 | 34.874 | 513-85-9 | 225936 |
| Hmf | 45.066 | 76330-16-0 | 237332 |
| Epifriedelanol Acetate | 31.182 | 131310 | 272944 |
| Corycavine | 67.178 | 521-87-9 | 276145 |
| Matrine | 63.775 | 519-02-8 | 285698 |
| Brusatol | 45.687 | 14907-98-3 | 299645 |
| Cis-Carveol | 45.611 | 1197-06-4 | 330573 |
| Marmesin | 50.277 | 13849-08-6 | 334704 |
| Gentianine | 54.666 | 439-89-4 | 354616 |
| Plenolin | 68.262 | 34257-95-9 | 354735 |
| Phyllanthin | 33.310 | 10351-88-9 | 358901 |
| Gentianidine | 54.445 | 2202-12-2 | 362908 |
| Piperitenone | 40.054 | 491-09-8 | 381152 |
| Naringenin | 59.294 | 480-41-1\|67604-48-2 | 439246 |
| Taxifolin | 57.842 | 480-18-2 | 439533 |
| D-2-Aminobutyrate | 68.778 | 2623-91-8 | 439691 |
| Ram | 50.500 | 10030-85-0 | 439710 |
| Endo-Fenchol | 76.426 | 2217-02-9 | 439711 |
| 2-Hydroxymethylserine | 95.700 | 17149-11-0 | 439893 |
| Pelargonidin | 37.988 | 134-04-3 | 440832 |
| (-)-Comphene | 34.979 | 79-92-5 | 440966 |
| (-)-Nopinene | 44.835 | 18172-67-3 | 440967 |
| (R)-Norcoclaurine | 82.543 | 106032-53-5 | 440988 |
| Beta-D-Apiose | 118.530 | 639-97-4 | 441474 |
| Pectin | 40.390 | 14982-50-4 | 441476 |
| Hippeastrine | 51.650 | 30803-79-3 | 441594 |
| Silandrin | 64.140 | 70815-32-6 | 441663 |
| Hypaconitine | 31.388 | 6900-87-4 | 441737 |
| Schottenol | 37.423 | 521-03-9 | 441837 |
| 5-O-Methylvisamminol | 37.990 | 80681-42-1 | 441970 |
| Physovenine | 106.214 | 6091-05-0 | 442113 |
| Decursin | 39.267 | 5928-25-6 | 442126 |
| Pinguisone | 66.068 | 22489-40-3 | 442388 |
| C09747 | 37.277 | 10-24-2 | 442408 |
| Neoastilbin | 40.543 | 29838-67-3 | 442437 |
| (+)-Iridodial Lactol | 93.827 | 550-45-8 | 442438 |
| Pedunculagin | 37.814 | 7045-42-3 | 442688 |
| 4-Hydroxyhomopterocarpin | 48.407 | 61135-95-3 | 442792 |
| Kadsurin A | 56.825 | 99340-07-5 | 442885 |
| Yangambin | 57.525 | 13060-14-5 | 443028 |
| Chebi:7 | 45.198 | 498-15-7 | 443156 |
| (R)-Linalool | 39.804 | 126-91-0 | 443158 |
| (L)-Alpha-Terpineol | 48.798 | 10482-56-1 | 443162 |
| Oxysanguinarine | 46.972 | 548-30-1 | 443716 |
| Cis-Dihydroquercetin | 66.437 | 480-18-2 | 443758 |
| Arachidonic Acid | 45.573 | 506-32-1\|93444-49-6 | 444899 |
| Zoomaric Acid | 35.776 | 373-49-9 | 445638 |
| Oleic Acid | 33.128 | 112-80-1 | 445639 |
| Ferulic Acid | 40.434 | 1135-24-6 | 445858 |
| Pomolic Acid Acetate | 32.295 | 35286-61-4 | 457300 |
| Poriferast-5-En-3Beta-Ol | 36.914 | 83-46-5 | 457801 |
| Isoarnebin 4 | 64.793 | 517-88-4 | 479503 |
| 2-Oxobutanol | 63.803 | 5077-67-8 | 521300 |
| Dihydrokaempferide | 50.562 | 3570-69-2 | 586387 |
| Cinnamaldehyde | 31.990 | 104-55-2 | 637511 |
| P-Coumaric Acid | 43.290 | 7400-08-0 | 637542 |
| Supraene | 33.546 | 111-02-4 | 638072 |
| 1-Acetyl-Beta-Carboline | 67.123 | 50892-83-6 | 638667 |
| P-Methoxycinnamaldehyde | 59.638 | 24680-50-0 | 641294 |
| 3-Hexenal | 50.335 | 4440-65-7 | 643139 |
| Nerol | 35.657 | 106-25-2 | 643820 |
| (2R)-5,7-Dihydroxy-2-Phenylchroman-4-One | 46.077 | 480-39-7 | 667544 |
| Methyl Caffeate | 30.684 | 3843-74-1 | 689075 |
| (-)-Taxifolin | 60.506 | 480-18-2 | 712316 |
| Dimethylcaffeic Acid | 45.832 | 14737-89-4 | 717531 |
| Isoferulic Acid | 50.826 | 25522-33-2\|537-73-5 | 736186 |
| Scopine | 49.821 | 498-45-3 | 1274465 |
| Ferulic Acid (Cis) | 54.965 | 1014-83-1 | 1548883 |
| Cis-Resveratrol | 41.127 | 501-36-0 | 1548910 |
| Cis-Jasmone | 32.073 | 488-10-8 | 1549018 |
| Neryl Acetate | 57.466 | 141-12-8 | 1549025 |
| Cis-P-Coumarate | 45.982 | 4501-31-9 | 1549106 |
| Caffeate | 54.971 | 331-39-5 | 1549111 |
| Cinnamic Acid, 3,4-Dimethoxy- (8Ci) | 63.862 | 2316-26-9 | 1585026 |
| Farnesylacetone | 37.842 | 1117-52-8\|762-29-8 | 1711945 |
| Nerylacetone | 45.528 | 3879-26-3 | 1713001 |
| Caryophyllene Oxide | 45.754 | 1139-30-6 | 1742210 |
| (2R)-Butan-2-Amine | 59.080 | 13952-84-6 | 2724537 |
| (S)-Matsutake Alcohol | 40.108 | 3391-86-4 | 2724898 |
| (2S)-Heptan-2-Amine | 46.442 | 123-82-0 | 2734517 |
| Hyoscine | 49.840 | 138-12-5 | 3000322 |
| Stigmast-7-Enol | 37.423 | 481-19-6 | 3080632 |
| Atractylone | 41.101 | 6989-21-5 | 3080635 |
| Leucodelphinidin | 43.448 | 55068-67-2 | 3081374 |
| Gbgb | 45.577 | 29307-60-6 | 3082301 |
| Thalifendine | 44.411 | 18207-71-1 | 3084288 |
| T-Muurolol | 30.414 | 19912-62-0 | 3084331 |
| Embinin | 33.912 | 52589-13-6 | 3085014 |
| Mamanine | 53.069 | 60394-92-5 | 3085182 |
| Quercetin | 46.433 | 117-39-5 | 5280343 |
| Formononetin | 69.674 | 485-72-3 | 5280378 |
| Acacetin | 34.974 | 480-44-4 | 5280442 |
| Luteolin | 36.163 | 491-70-3 | 5280445 |
| Calycosin | 47.752 | 20575-57-9 | 5280448 |
| Beta-Carotene | 37.184 | 7235-40-7 | 5280489 |
| Moupinamide | 86.712 | 66648-43-9 | 5280537 |
| Herbacetin | 36.074 | 527-95-7 | 5280544 |
| Stigmasterol | 43.830 | 83-48-7 | 5280794 |
| 3-Methylkempferol | 60.163 | 1592-70-7 | 5280862 |
| Kaempferol | 41.882 | 520-18-3 | 5280863 |
| Gla | 47.944 | 26566-61-0 | 5280933 |
| Linolenic Acid | 45.009 | 463-40-1 | 5280934 |
| Crocetin | 35.296 | 27876-94-4 | 5281232 |
| Flavoxanthin | 60.413 | 512-29-8 | 5281238 |
| Rubixanthin | 47.257 | 3763-55-1 | 5281252 |
| Spinoside A | 39.967 | 524-40-3 | 5281325 |
| Spinasterol | 42.979 | 481-18-5 | 5281331 |
| Harman | 33.096 | 486-84-0 | 5281404 |
| Enhydrin | 40.563 | 33880-85-2 | 5281441 |
| Arnebinol | 56.663 | 87064-17-3 | 5281558 |
| Azaleatin | 54.279 | 529-51-1 | 5281604 |
| Genkwanin | 37.130 | 437-64-9 | 5281617 |
| Dinatin | 30.972 | 1447-88-7 | 5281628 |
| Gentisein | 67.573 | 529-49-7 | 5281635 |
| Isorhamnetin | 49.604 | 480-19-3 | 5281654 |
| Norswertianin | 92.143 | 22172-15-2 | 5281658 |
| Morin | 46.230 | 480-16-0 | 5281670 |
| Tamarixetin | 32.859 | 603-61-2 | 5281699 |
| Wogonin | 30.685 | 632-85-9 | 5281703 |
| Coumestrol | 32.487 | 479-13-0 | 5281707 |
| Beta-Asarone | 35.612 | 5273-86-9 | 5281758 |
| Irolone | 46.867 | 41653-81-0 | 5281779 |
| Futoquinol | 59.834 | 28178-92-9 | 5281817 |
| Ellagic Acid | 43.065 | 476-66-4 | 5281855 |
| 2-Octenic Acid | 43.487 | 1871-67-6 | 5282713 |
| Gondoic Acid | 30.703 | 5561-99-9 | 5282768 |
| Odd | 41.700 | 2420-44-2 | 5282800 |
| Heptenal | 37.160 | 2463-63-0 | 5283316 |
| Eld | 31.196 | 181057-55-6 | 5283387 |
| 24-Methylidenelophenol | 44.193 | 1176-52-9 | 5283640 |
| Codeine | 45.479 | 76-57-3 | 5284371 |
| Methyl Linoleate | 41.934 | 112-63-0 | 5284421 |
| Myristelaidic Acid | 38.480 | 544-64-9? | 5312402 |
| Corydalmine | 52.501 | 30413-84-4 | 5316093 |
| Croweacin | 53.278 | 484-34-4 | 5316141 |
| Doederleinic Acid | 69.210 | 171596-14-8 | 5316957 |
| Linarin | 39.844 | 480-36-4 | 5317025 |
| Erythraline | 49.177 | 466-77-3 | 5317205 |
| Ethyl Caffeate | 103.851 | 66648-50-8 | 5317238 |
| Alkannan | 74.877 | 517-90-8 | 5317321 |
| Flavocommelin | 32.584 | 16049-42-6 | 5317357 |
| Flemiphilippinin C | 47.662 | 133830-92-9 | 5317360 |
| Gentiatibetine | 62.591 | 26005-36-7 | 5317559 |
| 2-Hexenol | 64.536 | 114411-82-4 | 5318042 |
| Isofraxidin | 39.746 | 486-21-5 | 5318565 |
| Kushenin | 47.622 | 99119-73-0 | 5318889 |
| Oroxylin A | 41.368 | 480-11-5 | 5320315 |
| Artemetin | 49.551 | 479-90-3 | 5320351 |
| Pectolinarigenin | 41.166 | 520-12-7 | 5320438 |
| Rhamnazin | 47.141 | 552-54-5 | 5320945 |
| Atractylenolide I | 37.365 | 73069-13-3 | 5321018 |
| Atractylodin | 44.493 | 55290-63-6 | 5321047 |
| 5,7,2',6'-Tetrahydroxyflavone | 37.013 | 82475-00-1 | 5321865 |
| Thalrugosaminine | 31.893 | 22226-73-9 | 5321919 |
| Torachrysone | 51.065 | 64032-49-1 | 5321977 |
| 5,2',6'-Trihydroxy-7,8-Dimethoxyflavone | 45.047 | 92519-93-2 | 5322059 |
| 5,7,4'-Trihydroxy-6-Methoxyflavanone | 36.627 | 94942-49-1 | 5322074 |
| 5,7,4'-Trihydroxy-8-Methoxyflavone | 36.562 | 57096-02-3 | 5322078 |
| Robinin | 39.844 | 301-19-9 | 5351997 |
| 2-Ethylhexenal | 37.077 | 128744-21-8 | 5354264 |
| Ethyl Oleate (Nf) | 32.397 | 111-62-6 | 5363269 |
| Methyl Vaccenate | 31.898 | 52380-33-3 | 5364432 |
| Exceparl M-Ol | 31.898 | 139152-82-2 | 5364509 |
| Damascenone | 36.427 | 23726-93-4 | 5366074 |
| Cis-Zimtsaeure | 38.187 | 621-82-9 | 5372954 |
| Flazin | 94.276 | 100041-05-2 | 5377686 |
| Taraxerol | 38.403 | 22076-46-6 | 5458935 |
| Aldehydo-L-Arabinose | 31.885 | 147-81-9 | 5460291 |
| Bergaptin | 41.730 | 7380-40-7 | 5471349 |
| Iristectorigenin (9Ci) | 71.548 | 37744-62-0 | 5488781 |
| Iristectorigenin A | 63.364 | 39012-01-6 | 5491637 |
| Senkirkine | 56.156 | 2318-18-5 | 6305177 |
| Ethyl Linolenate | 46.101 | 1191-41-9 | 6371716 |
| (Z)-Caryophyllene | 30.291 | 87-44-5 | 6429301 |
| (7Ar)-4,4,7A-Trimethyl-6,7-Dihydro-5H-Benzofuran-2-One | 40.478 | 15356-74-8 | 6432173 |
| Isomenthol | 55.304 | 3623-52-7 | 6432468 |
| Raphanin | 61.472 | 592-95-0 | 6433206 |
| Butenylisothiocyanate | 66.921 | 34424-44-7 | 6439701 |
| Yuanhuacin | 31.832 | 60195-70-2 | 6440205 |
| Yuanhuadine | 30.731 | 76402-66-9 | 6440572 |
| Ajugacumbin F | 70.692 | 132922-44-2 | 6442776 |
| Tigloidine | 30.457 | 495-83-0 | 6540490 |
| (S)-2-Aminobutanoate | 61.337 | 80-60-4 | 6857370 |
| Scropolioside D | 36.617 | 148000-43-5 | 6918700 |
| Gup | 43.043 | 7322-31-8 | 6971098 |
| Chebi:39932 | 32.794 | 3391-86-4 | 6992244 |
| [(3S)-3,7-Dimethylocta-1,6-Dien-3-Yl] Acetate | 36.843 | 115-95-7 | 6999980 |
| Glucobrassicin | 53.640 | 4356-52-9 | 9601691 |
| (3S,5R,8R,9R,10S,14S)-3,17-Dihydroxy-4,4,8,10,14-Pentamethyl-2,3,5,6,7,9-Hexahydro-1H-Cyclopenta[A]Phenanthrene-15,16-Dione | 43.556 | 139954-00-0 | 9841735 |
| Smitilbin | 37.603 | 222846-33-5 | 9889962 |
| Aurantiamide Acetate | 58.381 | 56121-42-7 | 10026486 |
| 5-(2-Hydroxyethyl)-2-Methoxyphenol | 31.950 | 149596-95-2 | 10034991 |
| Anhydrobelachinal | 43.574 | 219827-03-9 | 10742927 |
| Alloaromadendrene | 54.138 | 25246-27-9 | 10899740 |
| (1S,5S)-1-Isopropyl-4-Methylenebicyclo[3.1.0]Hexane | 46.205 | 87-41-5 | 11051711 |
| (1S,4R)-1,7,7-Trimethylbicyclo[2.2.1]Hept-2-Ene | 39.619 | 464-17-5 | 11062489 |
| 1-[(2S)-Oxolan-2-Yl]Ethanone | 31.284 | 131328-27-3 | 11170944 |
| Procyanidin B1 | 67.873 | 20315-25-7 | 11250133 |
| Danshensu | 36.915 | 76822-21-4 | 11600642 |
| (6Z,10E,14E,18E)-2,6,10,15,19,23-Hexamethyltetracosa-2,6,10,14,18,22-Hexaene | 33.546 | 7683-64-9 | 11975273 |
| Silydianin | 59.654 | 29782-68-1 | 11982272 |
| Cepharamine | 56.472 | 15444-26-5 | 12302744 |
| Istanbulin-A | 80.101 | 35481-83-5 | 12304519 |
| Cycloartenone | 40.570 | 511-63-7 | 12305360 |
| Cirsiumaldehyde | 41.385 | 7389-38-0 | 12366272 |
| Sophoranol | 67.319 | 3411-37-8 | 12442899 |
| Saringosterol | 43.476 | 6901-60-6 | 14161394 |
| Arnicolide C | 76.915 | 34532-67-7 | 14656909 |
| Isosilychristin | 30.315 | 77182-66-2 | 14849116 |
| Aeginetic Acid | 48.315 | 53537-92-1 | 15693867 |
| Rugosin A | 38.259 | 84744-48-9 | 16132354 |
| Longifolene | 39.495 | 475-20-7 | 16396350 |
| Neohesperidin | 57.441 | 13241-33-3 | 17751030 |
| Worenine | 45.833 | 38763-29-0 | 20055073 |
| Cryptogenin | 35.114 | 468-99-5 | 21117640 |
| Linoleyl Acetate | 42.101 | 5999-95-1 | 21159087 |
| Chrysanthemaxanthin | 38.724 | 26989-20-8 | 21160900 |
| Sophojaponicin | 41.506 | 30142-39-3 | 44257440 |
| Abrectorin | 32.165 | 76575-03-6 | 44257585 |
| Leucanthoside | 32.116 | 6980-25-2 | 44258368 |
| Niloticin | 41.414 | 115404-57-4 | 44559946 |
| Indicumenone | 35.681 | 109063-92-5 | 46173956 |
| Sarcostin | 33.029 | 18607-76-6 | 46173994 |
| Precatorine | 85.061 | 36675-57-7 | 54704420 |
| Digallate | 61.849 | 536-08-3 | 54711004 |
| Isocorynoline | 47.439 | 475-67-2 | 56842002 |
| Xilingsaponin A | 39.043 | 41059-79-4 | 71306914 |
| Chebulic Acid | 71.998 | 23725-05-5 | 71308174 |
| Yadanzioside P | 58.757 | 79439-84-2 | 73345262 |
| Yadanzioside I | 61.131 | 99132-95-3 | 73817507 |
| Yadanzioside L | 31.365 | 99132-97-5 | 73817508 |
| Trans-2,4-Decadienal | 51.028 | 30551-18-9 | 86736649 |
| Belamcandal | 30.072 | 138501-57-2 | 101615675 |
| Yadanzioside H | 62.770 | 95258-21-2 | 101659162 |
| Yadanzioside J | 38.697 | 99132-96-4 | 101659163 |
| Furosin | 40.530 | 81552-37-6 | 101921699 |
| Isoengelitin | 34.651 | 30987-58-7 | 101937309 |
| Emblicanin B | 37.080 | 180465-45-6 | 119058017 |
| Acetylshikonin | 62.388 | 24502-78-1 | 131674214 |
| Curdione | 38.943 | 13657-68-6 | 131675179 |
| Scopolamine | 67.974 | 51-34-3\|64069-66-2 | 134767951 |
| Vulgaxanthin-I | 56.140 | 904-62-1 | 135438599 |
| Santalol | 40.174 | 11031-45-1 | 137947084 |
| Mangiferolic Acid | 36.159 | 4184-34-3 | 145999601 |
| Ceanothic Acid | 33.412 | 21302-79-4 | 146158060 |
| Cycloartenol | 38.686 | 469-38-5 | 146158953 |
